# Digital behavioral dietary interventions to promote a healthy diet among children and adolescents: a scoping review of technologies, design, behavioral theory, and assessed outcomes

**DOI:** 10.1080/21642850.2024.2430965

**Published:** 2024-11-29

**Authors:** Zoë van der Heijden, Desiree Lucassen, Janine Faessen, Guido Camps, Yuan Lu, Henk Schipper, Sanne Nijhof, Elske Brouwer-Brolsma

**Affiliations:** a Division of Human Nutrition and Health, Department Agrotechnology and Food Sciences, Wageningen University and Research, Wageningen, The Netherlands; b Department of Industrial Design, Eindhoven University of Technology, Eindhoven, The Netherlands; c Department of Pediatric Cardiology, Erasmus MC: Sophia’s Children Hospital, Rotterdam, The Netherlands; d Department of General Pediatrics, UMC Utrecht, Utrecht, The Netherlands

**Keywords:** Scoping review, child nutrition, behavior change interventions, digital technologies

## Abstract

**Background:**

Childhood overweight and obesity prevalence steeply increased during recent decades, prompting the development of many digital behavioral dietary interventions (DBDIs). However, a coherent overview is lacking, which is crucial for delineating research in this field.

**Objective:**

This scoping review outlines the landscape of DBDIs for improving dietary behaviors in children and adolescents, including delivery modes, design and development approaches, behavioral theory, and outcomes assessed. Secondary objectives involved examining the integration of behavior change techniques (BCTs) and identifying outcomes favoring DBDIs.

**Methods:**

Following PRISMA guidelines, PsycInfo, PubMed, and Scopus were systematically searched for evaluated DBDIs. Two reviewers independently screened titles and abstracts; one performed full-text screening. Studies included had a digital component, targeted dietary behavior, focused on children or adolescents, and evaluated effects on behavior change, health, or process evaluation outcomes. One reviewer extracted data, including general information, theoretical underpinning, and outcomes assessed, while BCTs were coded independently by two reviewers. DBDIs were deemed favorable if significant improvements were observed in all outcomes (*p* ≤ .05).

**Results:**

From 51 included studies, 41 DBDIs were identified, including app-based (37%), web-based (29%), computer-based (27%), text-message-based (5%), and combined technology tools (2%). Stakeholders were involved in the design of 59% of DBDIs, with 5% using co-design methodologies. Studies evaluated behavior change outcomes (86%), process evaluation outcomes (59%), and health outcomes (20%). DBDIs included an average of 6.2 BCTs, primarily ‘Feedback on behavior’ (56%) and ‘Non-specific reward’ (46%). Among experimental studies, 15% yielded favorable results, 58% mixed results, and 28% no favorable results.

**Discussion:**

This review outlines the diverse landscape of DBDIs, highlighting various technological delivery modes and outcomes assessed. Methodological variations and limitations challenge consistent effectiveness assessment. Future research should prioritize rigorous study designs to understand efficacy and identify effective BCTs among diverse pediatric populations. Leveraging co-design methods may enhance engagement and effectiveness.

## Introduction

Healthy dietary habits are key in weight management and the prevention of later-life diseases, e.g. type 2 diabetes, cardiovascular disease, and several cancer types (Di Cesare et al., 2019; Jebeile et al., 2022). Nevertheless, most children and adolescents do not meet recommended national dietary guidelines (Hardy et al., 2017; Kovács et al., 2014), often adopting unhealthy eating habits. To illustrate, 4-to-12-year-old children in the Netherlands eat on average 74 g of vegetables a day while the recommended daily intake is to eat at least 125–175 g per day (van Rossum et al., 2017). Moreover, Dutch children have one of the highest intakes of sugar-sweetened beverages in Europe (Brug et al., 2012), known to contribute to weight gain in children (Malik et al., 2013). As dietary habits adopted in childhood preserve into adulthood and thereby shape long-term health outcomes (Craigie et al., 2011), addressing this issue is paramount (WHO, 2018).

Over the past decades, numerous interventions aimed to encourage healthier dietary habits in children, often through educational programs, nutritional counseling, or changes to school meals (Pereira & Oliveira, 2021; Shorey & Chan, 2021). So far, most provided generic advice rather than tailored recommendations (Al-Awadhi et al., 2021; Lucassen et al., 2021), limiting their effectiveness in promoting sustained behavior change (Pereira & Oliveira, 2021; Shorey & Chan, 2021). Personalized interventions improve dietary intake more effectively than generic advice (Jinnette et al., 2021), but may still be insufficient for long-term behavior change. Co-design, involving end-users in the design process, can enhance satisfaction and engagement by catering to their unique needs and wishes (Slattery et al., 2020). However, understanding of co-design methods and their effectiveness in health interventions is limited. Additionally, the implementation of these face-to-face personalized and co-designed interventions is costly and time-intensive (Koh et al., 2021), limiting scalability.

In the coming decades, novel digital behavioral dietary interventions (DBDIs) using tools such as smartphone applications, wearable devices, online platforms, and online games can play a key role in providing effective dietary interventions. Digitization already led to tools for automated food intake registration (Eldridge et al., 2019; Threapleton et al., 2022), enabling its integration into newly developed interventions for more individual-tailored dietary advice (Threapleton et al., 2022). Another example includes an interactive nutrition education module using gamification to engage users and provide personalized feedback and recommendations om healthy eating (Ghammachi et al., 2022). While these digital interventions may be more effective for children (Arias-de la Torre et al., 2020), little is known about the specific age groups and socio-demographic backgrounds they target.

By leveraging digital platforms and automated features, DBDIs can provide adaptive, tailored approaches to a broad audience at relatively low costs, addressing individuals contexts, needs, and preferences (Stanczyk et al., 2014). Integrating behavior change theories, such as Social Cognitive Theory or Self-Determination Theory, enhances effectiveness by providing a framework for understanding and influencing behavior (Webb et al., 2010). These theories can be translated into behavior change techniques (BCTs) (Michie et al., 2013), such as goal setting, information provision, or increasing social support, to target underlying factors of dietary behaviors (Martin et al., 2013). Understanding these theories and their application through BCTs is crucial for advancing DBDIs, helping achieve dietary goals, providing relevant information, and fostering social networks to support behavior change.

Numerous reviews have examined DBDIs for dietary support in pediatric populations, but often focus narrowly on specific patient groups (Antwi et al., 2013; Arthurs et al., 2022; Badawy & Kuhns, 2017; Brown et al., 2022; Burrows et al., 2015; De Sousa et al., 2022; Dute et al., 2016; Loescher et al., 2018; Partridge et al., 2020; Quelly et al., 2016; Tully et al., 2020; Yau et al., 2022; Yien et al., (2021)), delivery modes (e.g. type of DBDI) (Adaji, 2022; Antwi et al., 2013; Arthurs et al., 2022; Badawy & Kuhns, 2017; Brown et al., 2022; Burrows et al., 2015; Chow et al., 2020; De Sousa et al., 2022; Dute et al., 2016; Hamel & Robbins, 2013; Loescher et al., 2018; Partridge et al., 2020; Quelly et al., 2016; Schoeppe et al., 2016; Schoeppe et al., 2017; Suleiman-Martos et al., 2021; Yau et al., 2022) and/or outcomes assessed (e.g. weight, usability, or acceptability) (Arthurs et al., 2022; Badawy & Kuhns, 2017; Burrows et al., 2015; Partridge et al., 2020; Quelly et al., 2016; Tully et al., 2020; Yien et al. ; Oh et al., 2022), resulting in a fragmented field. While specific reviews offer quantitative answers to focused questions, they may overlook broader trends, common gaps, and methodological variations. A comprehensive review is crucial to pinpoint knowledge gaps, direct future research, and provide a consolidated and accessible source of information on DBDIs for children and adolescents. Moreover, few reviews have explored BCTs in health behavior change interventions (Ashton et al., 2019; Brannon & Cushing, 2015; Brown et al., 2022; Milne-Ives et al., 2020; Yau et al., 2022), particularly within DBDIs for children and adolescents, highlighting the need for a broader review to comprehensively understand study designs applied and BCTs applications across various contexts.

This paper presents a scoping review on DBDIs aimed at enhancing dietary behaviors in pediatric populations to address existing gaps. Scoping reviews are suitable for mapping how a topic has been studied (Arksey & O'malley, 2005), map discipline parameters (Armstrong et al., 2011), and summarizing findings from studies that are heterogeneous in methods or discipline (Tricco et al., 2018). The primary aim is to provide a comprehensive overview of available DBDIs for children and adolescents, including technological delivery modes (e.g. apps), design and development approaches (e.g. stakeholder involvement and target populations), behavioral theory (e.g. behavior change techniques), and outcomes assessed (i.e. outcome categories health, behavior change, and process evaluation outcomes). Secondary objectives involve examining BCT integration across DBDIs and evaluating assessed outcomes for significant improvements. Addressing this gap is vital for establishing effective interventions, replicating studies, and advancing implementation in healthcare.

## Materials and methods

### Literature search

This scoping review followed the PRISMA checklist for scoping reviews (Supplemental Online Material 1) (Tricco et al., 2018), and was developed with assistance from a research librarian. The systematic search, conducted in April 2022 and updated in September 2022, included Scopus, PsycInfo, and PubMed databases. Medical Subject Heading included terms, synonyms, and keywords relating to four categories: (1) children and adolescents, (2) modes of delivery (computer-, smartphone-, tablet-, internet-, chatbot-, or game-based), (3) health outcomes (nutrition behavior, physical activity, sedentary behavior, obesity indices, or clinical health outcomes), and (4) assessing behavior change outcomes, (clinical) health outcomes, biochemical health outcomes, or process evaluation outcomes. We did not mandate behavior change theory and BCTs for inclusion in DBDIs. This allowed us to objectively assess how often these interventions are grounded in behavioral theory and use specific BCTs, without biasing the sample. The search strategy, detailed in Supplemental Online Material 2, included at least one search term from each of the four categories mentioned and was used to retrieve the publications included in this review. Initially, we aimed to encompass a broad range of health behaviors, including physical activity, and clinical outcomes. However, after title and abstract screening, we narrowed the focus to nutrition-related studies, to align with our core expertise. After completion of full-text screening, citation searches and reference lists of included papers were cross-checked for additional relevant articles. No registered protocol is available, but the search strategy can be found in Supplemental Online Material 2.

### Eligibility criteria

Given fast-paced evolutions of DBDIs, results were constrained to English-language articles published after 2010 to capture recent technological developments. A DBDI is defined as software available on any digital device (e.g. tablet, computer, smartphone) designed to promote healthy behaviors through automated technology-driven interventions. Included were articles *evaluating automated* (i.e. function independently of human interference) DBDIs providing immediate feedback on *dietary behavior change* for *healthy* children and adolescents (4–18 years). This age range covers a broad developmental spectrum from early childhood through adolescence. Eligible study types encompassed randomized controlled trials (RCTs), quasi-experimental studies (QES), and exploratory studies focusing on process evaluation outcomes, such as satisfaction, acceptability, usability, or experiments lacking a control group. Exploratory studies were excluded from the secondary analysis of the impact of DBDIs on behavior or health due to their differing objectives. Studies had to report at least one reported interaction with children and adolescents *as primary user*. We did not require the application of behavioral theory as an inclusion criterion, such as BCTs, to allow a comprehensive exploration of current DBDIs. Exclusions were articles on DBDIs targeting obese children (>85th percentile of age-appropriate BMI), those hospitalized or diagnosed with conditions affecting dietary intake (e.g. diabetes, eating disorders), and studies describing only the design or development of software/hardware prototypes without evaluation data, as well as commercial DBDIs.

### Selection process and data extraction

Two researchers screened titles, abstracts, and keywords from the databases, removing duplicates using EndNote 21. One researcher reviewed full texts of potentially relevant articles, with discrepancies resolved by a senior researcher. Additionally, after discussion within the research team, two articles targeting adolescents up to 21 years were included, expanding our initial planned age range. Extracted data covered *general information on DBDI* (e.g. name, mode of delivery, design methodologies applied, target populations, or DBDI behavioral goal), *theoretical underpinning* (e.g. behavioral theory applied and BCTs integrated), and *general study information* (e.g. year of publication, country, study design, study setting, sample characteristics, and intervention duration), and *outcomes assessed*. Extracted data on outcomes assessed included primary (i.e. main effects), secondary (i.e. supplementary effects), and other outcomes (i.e. process evaluation outcomes or exploratory outcomes). These outcomes were categorized into three main outcome categories: behavioral outcomes (e.g. food intake, physical activity, knowledge, psychosocial factors such as intention, perception, or attitude), health outcomes (e.g. weight and BMI), and process evaluation outcomes (e.g. acceptability, usability, or engagement).

#### Favorability of results

For RCTs and QES, excluding exploratory studies and other outcomes (i.e. process evaluation or exploratory outcomes), we assessed favorability of DBDIs based on reported outcomes, providing a proxy for intervention performance. Exploratory studies and other outcomes were excluded from this secondary analysis due to differing objectives and study designs, limiting comparability and impact assessment. DBDIs evaluated in RCTs or QES were classified as ‘*favorable*’ (significant improvements for all primary or secondary outcomes), ‘*some favorable outcomes’ (*significant improvements, but not for all outcomes or not maintained at follow-up, if applicable), and ‘*not favorable’* (no significant improvements). If an outcome included multiple sub-determinants (e.g. individual food items within the overarching measure of food intake), a DBDI was considered favorable if at least 50% of the sub-determinants showed significant improvements. A *p*-value of ≤ .05 was considered statistically significant. This classification system was specifically developed for this study to accommodate the diversity of outcomes and methodologies across the included studies. We also considered the duration of effects, with short-term assessed within one-month post-intervention, medium-term within three months, and long-term at six months or more.

#### BCT coding

Included DBDIs underwent coding for BCTs using the Behavior Change Technique Taxonomy v1 (BCTTv1), which comprises 93 BCTs in 16 clusters to specify, interpret, and implement intervention components for influencing behavior (Michie et al., 2013). BCTs have been coded specifically in this review due to their granular and actionable approach. BCTs were extracted from DBDI descriptions if authors explicitly mentioned them according to the BCTTv1 in the intervention. In cases without explicit BCT reporting, each DBDI description was assessed for the presence or absence of BCTs based on BCTTv1. Coding efforts focused on DBDIs where BCT use was not explicitly reported to avoid redundancy and streamline reporting. Two researchers, having completed the online BCT evaluation course for standardized coding procedures, independently coded the intervention descriptions, isolated from the included article, its supplemental material, or development paper, if applicable. Consultation ensured alignment after coding five DBDI descriptions, and discrepancies were resolved by consulting a third researcher when needed.

### Ethics approval

Not applicable, as this study does not involve human participants.

## Results

**Figure 1.**
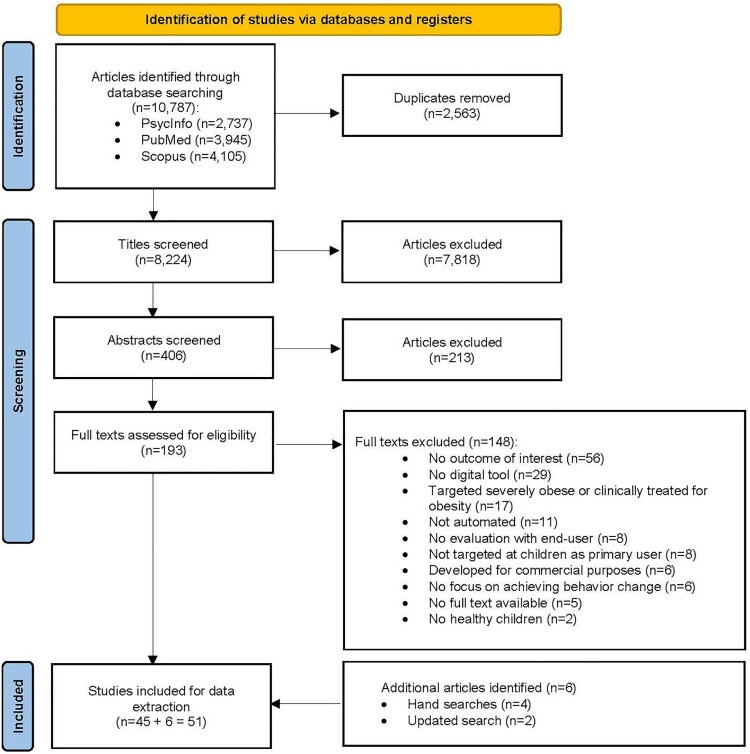
Preferred reporting items for scoping reviews and meta-analyses flow diagram summarizing the process of study selection.

The search resulted in 8224 articles with 193 selected for full-text review. Of these, 148 did not meet inclusion criteria, resulting in 51 studies describing 41 unique DBDIs for this review (Figure 1).

### Summary of included studies

**Figure 2.**
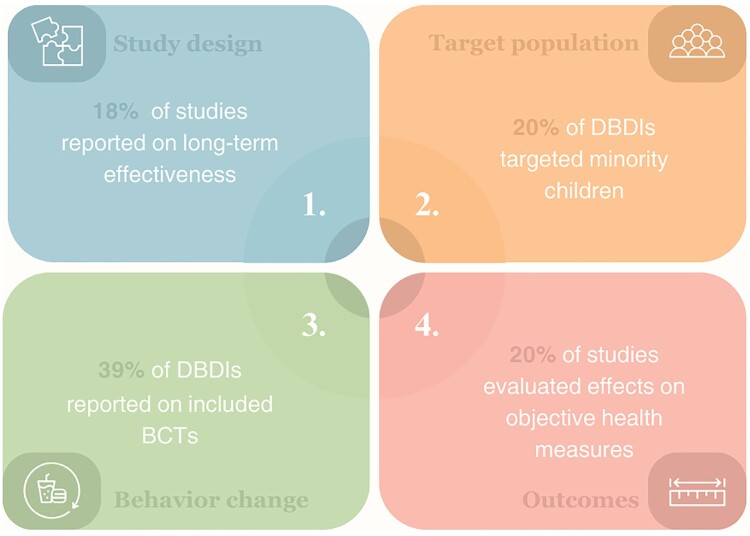
Characteristics of 41 digital behavioral dietary interventions and 51 (evaluation) studies to improve dietary behaviors in children and adolescents.

Table 1 provides an overview of study characteristics. A summary of study features is depicted in Figure 2.

**Table 1.** Summary of study characteristics.

First author (year), description	Mode of delivery	Aim/outcome (P, S, O)	Population (n)	Study design/duration	Key findings	Favorable outcomes
Ahn et al. (2016) *A virtual pet exergame (dog) as a vehicle for FV promotion among children.*	Computer-based	To evaluate efficacy of using virtual pets for promoting FV consumption *Food servings (P) and intake (P), preference (P)*	7–13 years, US (68)	*RCT*: 3 arms: IG (computer + dog), CG (only computer), or CG (no treatment)/*3 days*	IG was served more FV than both CGs but did not consume more FV. Food preferences did not differ between IG and CGs.	Some ^a^ P(1/3) ^b^ *Short-term* ^c^
Alblas et al. (2020) *Game in which users provide residents of a sinking island with healthy foods, to restore island’s buoyancy.*	Computer-based	To investigate its effects on implicit attitudes and snack choices *Attitudes (P), digital food intake (snack) (S)*	11–13 years, overweight, the Netherlands (79)	*QES:* IG played game. CG played identical game, but food pictures were replaced with pictures of fossil fuels./*40 min*.	No effects on implicit attitudes towards food and snack choice were found. Among participants with less healthy baseline attitudes, healthier posttest outcomes were found.	No P(0/1) ^b^ S(0/1) ^b^ *Short-term ^c^*
Baranowski et al. (2011), Baranowski et al. (2019), Wang et al. (2015) *Games in which the player helps unhealthy inhabitants escaping an oppressed country by maintaining a healthy lifestyle.*	Computer-based	To evaluate the effect on children's diet, PA, and adiposity *Food intake (P), anthropometry (P), PA (P), engagement (O)*	10–12 years, between 50th percentile and 95th percentile, US (133)	*RCT:* IG played Diab and Nano in sequence. CG played control games at popular websites/*6 h in 2 months*	IG increased FV consumption in all assessments. No changes found for PA, body composition, or water intake.	No P(0/3) ^b^ *Medium-term* ^c^
Bell et al. (2018) *Game in which users explore gardening and cooking, by growing FVs and preparing meals in a virtual garden for Ladybug Dotty.*	App-based	To examine its effect on food intake and psychosocial determinants *Food intake (P), attitude (self-efficacy, preference, motivation) (P), anthropometry (P)*	8–12 years, minority children, US (180)	*QES:* IG played game, received Virtual Sprouts curriculum, and family activity. No-intervention CG/*3 h over 3 weeks*	IG increased self-efficacy to eat and cook FVs score. No differences in intake or self-efficacy to garden were observed.	Some ^a^ P(1/3) ^b^ *Short-term* ^c^
Benavides et al. (2021) *App facilitating social interaction and personalized (push) notifications. The app also contains a public part.*	App-based	To assess its effectiveness on improving BMI *Anthropometry (P), dietary habits (P), PA (P), degree of centrality in social network, usage (O)*	11–13 years, Spain (301)	*QES:* app was used by IG + CG; CG had only access to public part/*(14 week, at least once per week).*	Use of app helped IG to achieve BMIs that were closer to 50th percentile. Diet scores and social networks improved among IG.	Yes P(3/3) ^b^ *Short-term ^c^*
Byrne et al. (2012) *Virtual pet game. Player receives prompt from pet to eat and photograph their breakfast, that they send to a server.*	App-based	To examine how a virtual pet influences children's likelihood to eat breakfast *Food intake (likelihood & healthiness) (P), self-efficacy (S), perception (S), attitudes (O), attachment (O), motivation (O)*	12–14 years, US (39)	*RCT:* 3 arms: positive and negative feedback condition, positive or neutral only feedback condition, or CG (no pet)/*9 days*	Participants receiving positive and negative feedback were more likely to eat breakfast, to be attached, and to be motivated to help their pet. No difference in self-efficacy.	Some ^a^ P(0/2) ^b^ S(1/2) ^b^ *Short-term* ^b^
Chamberland et al. (2017) *Platform to record daily consumption of FVs and milk (alternatives), that automatically summarizes individuals’ and team levels’ attempts. Provides rewards.*	Web-based	To evaluate its impact on promoting consumption of FV and milk alternatives and to identify facilitators and barriers for success *Food intake (FV & milk alternatives) (P), anthropometry (S), experience (O)*	13–14 years, Canada (282)	*RCT:* IG recorded consumption of FV and milk alternatives and participated in team challenges. CG accessed plain website to report intake./*6 weeks (daily use).*	IG reported increase of FV and milk alternatives consumption per day. Effect did not sustain at follow-up. Facilitators of success were (1) team aspect, (2) use of technology, and (3) recording results outside classroom hours.	Some ^a^ P(2/2) ^b^ S(0/1) ^b^ *Medium-term ^c^*
Chang et al. (2022) *Game in which children replenish the energy of a mouse. By eating healthy, mouse earns points and runs faster.*	Computer-based	To evaluate its effectiveness in improving knowledge and junk food intake *Knowledge (P), food intake (P), applicability (O)*	5–6 years, Taiwan (104)	*QES:* IG played game. No-intervention CG./*4 h in 4 weeks*	IG showed higher levels of nutritional knowledge than CG, but frequency of junk food intake did not differ.	Some ^a^ P(1/2) ^b^ *Short-term ^c^*
Chen et al. (2011) *Program to enhance self-efficacy, understanding, and problem-solving skills by providing knowledge, interactive digital cooking, and goal setting.*	Web-based	To examine its feasibility and efficacy in promoting healthy lifestyles *Anthropometry (P), food intake (P), PA (P), knowledge (PA & nutrition) (P), self-efficacy (PA & nutrition) (P)*	12–15 years, Chinese and Chinese American, US (50)	*RCT:* IG + parents participated in online sessions. CG received general information via control website*/8 weeks (weekly session)*	IG decreased waist-to-hip ratio and diastolic blood pressure, and increased FV intake, level of PA, and knowledge about PA and nutrition.	Some ^a^ P(1/5) ^b^ *Long-term ^c^*
Cullen et al. (2013) *Website presenting information materials, a nutrition calculator to identify and set behavioral goals, role model videos, recipes, and blogs.*	Web-based	To evaluate its impact on healthy eating and PA behaviors *Food intake (P), PA (P), availability (PA & diet) (S), self-efficacy (PA & diet) (S), engagement (PA) (S), safety (PA) (S), experience (O)*	12–17 years, US (284)	*RCT:* IG used intervention website. CG website was constructed from intervention website, but behavioral change features were removed./*8 weeks.*	IG reported eating 3 or more daily vegetable servings in the past week compared to CG. Both groups reported increases in PA and decreases in sedentary behavior.	Some ^a^ P(0/2) ^b^ S(1/6) ^b^ *Short-term* ^c^
De Cock et al. (2018) *Game in virtual high school. Contains a snack track tool, a credit system, goal-setting booklet and report card.*	App-based	To evaluate its feasibility and impact on snack intake *Food intake (snack) (P), awareness (S), intentions (S), attitudes (health & taste) (S), self-efficacy (S), knowledge (S), habits (S) exposure (O), acceptability (O), reach (O)*	14–16 years old, Belgium (988)	*QES:* IG used app. CG continued usual school curriculum/*4 weeks*.	No effects on snack ratio or other outcomes were found. Exposure to intervention was not associated with positive intervention effects. Satisfaction ratings were low in both high- and low-users.	No P(0/1) ^b^ S(0/7) ^b^ *Short-term ^c^*
dos Santos Chagas et al. (2020) *Card game presenting the concept of an adequate and healthy diet. The cards in the game represent different foods, characters, and habits*	App-based	To assess its impact on food consumption, knowledge, and self-efficacy in adopting healthier habits *Perception (P), dietary practices (P), knowledge (P), self-efficacy (P)*	13–19 years, Brazil (318)	*RCT:* IG was instructed to play the game. No-intervention CG./*7-17 days*.	IG reported reductions on eating while watching TV/studying, having fast food meals, and showed increased self-efficacy in adopting healthy eating practices.	Some ^a^ P(1/4) ^b^ *Short-term ^c^*
Espinosa-Curiel et al. (2020) *Game in which players, being secret agent, review food menus of restaurants to save children from an evil chef.*	Computer-based	To design and test FoodRateMaster *Knowledge (O), food intake (O), parent perception (O)*	8–10 years, Mexico (60)	*E:* Interdisciplinary team developed game. Game was tested in individual gaming sessions /*3 h in 6 weeks*	IG increased food knowledge, self-reported frequency of some healthy foods, and decreased intake of some unhealthy foods. Parents indicated that children’s eating attitudes were positively influenced.	N.a.
Espinosa-Curiel et al. (2022) *Game in which players, being secret agent, take care of a group of children whose memories on healthy lifestyle behaviors have been erased. These need to be activated again.*	Computer-based, exergame	To describe design and development and evaluate its feasibility, acceptability, and preliminary efficacy *Knowledge (P), intentions to conduct healthy behaviors (S), food intake (S), feasibility (O), acceptability (O)*	8–11 years, Mexico (40)	*QES:* IG played game and CG received a behavioral education talk about healthy lifestyles. /*3 h in 4 weeks.*	Knowledge scores and intention to perform increased for all health behaviors in IG. IG reported reduced intake of some unhealthy foods. Game was well received and was feasible and acceptable.	Yes *P*(1/1) ^b^ S(2/2) ^b^ *Short-term ^c^*
Ezendam et al. (2012) *Program that provides information, self-assessments, feedback, goal setting, and action-planning for improving PA, sedentary, and dietary behaviors.*	Web-based	To evaluate short- and long-term results on PA, decrease sedentary behavior, and healthy eating *Food intake (P), PA (P), sedentary behavior (P), anthropometry (P)*	12–13 years, the Netherlands (883)	*RCT:* Schools were randomized in IG or no-intervention CG/*2 h in 10 weeks*	Positive short-term effects on diet were found for IG, but no effects on PA and sedentary behavior.	No P(0/4) ^b^ *Long-term ^c^*
Farrow et al. (2019) *Game in which children practice mathematical skills, while being exposed to images of V. V can be fed to character.*	App-based	To evaluate its effectiveness in increasing children's acceptance and liking of V *Food intake (V) (P), liking (V) (P)*	3–6 years, UK (74)	*QES:* IG played with app, CG played with control game Turtle Maths.*/10 min*	IG ate more vegetables and increased their liking for these vegetables. No effects were in CG.	Yes P(2/2) ^b^ *Short-term ^c^*
Folkvord et al. (2021) *Game in which Garfield needs to make several cities healthy again, by playing several nutrition-related minigames.*	App-based	To examine its effect of on eating behavior and attitudes toward healthy and unhealthy foods *Attitude (food and game) (P), food intake (P)*	8–12 years, the Netherlands (157)	IG played game in class. No-intervention CG./*1 week*.	No effect on attitude towards fruit, snacks, or on consumption of fruits or snacks was found in IG.	No *P*(0/2) ^b^ *Short-term ^c^*
Brown et al. (2020); Froome et al. (2020) *Game in which residents of a town have robots to help making healthier dietary choices.*	App-based	To describe its iterative development and user testing *Engagement (O), usability (O)*	9–12 years, Canada (92)	*E:* user testing was performed for every game module/*20-30 min*	IG wanted to play game again, found app easy-to-use and fun, and goals clearly defined.	N.a.
		To determine efficacy in improving children's knowledge of Canada's Food Guide *Knowledge (P), knowledge per subgroup (S)*	8 – 10 years, Canada (73)	*RCT:* IG played game, CG played control game My Salad Shop Bar./*50 min in 5 days*	IG showed increases in nutrition knowledge and for subgroups FV, protein food, and whole grain foods. No differences for drinks sub-score.	Some ^a^ *P*(1/1) ^b^ S(2/4) ^b^ *Short-term ^c^*
Hermans et al. (2018) *Educational game on how to read nutritional profiles and main functions of macronutrients.*	Computer-based, exergame	To test short-term effectiveness in improving knowledge and food intake *Knowledge (P), food intake (S)*	8–12 years, the Netherlands (108)	*QES*: IG played game twice on two days. CG played Super Shopper/*1 h in 2 weeks*	IG showed better nutritional knowledge immediate post-test. No differences in food intake.	No ^a^ P(0/1) ^b^ S(0/1) ^b^ *Short-term ^c^*
Kato-Lin et al. (2020) *Game in which players fight against robots representing unhealthy foods. Body shape of avatar changes because of unhealthy food choices.*	App-based	To investigate its immediate impact on food choices *Food intake (P), game patterns (O)*	10–11 years, India (104)	*RCT:* IG played game, CG played board game Uno./*40 min in 1 week*	IG increased identified and chosen healthy foods. Variation was found in play patterns.	Yes P(1/1) ^b^ *Short-term ^c^*
Mack et al. (2020) *Game about a competition between 2 villages to regain knowledge on nutrition and a healthy lifestyle by incorporating competitive elements.*	Computer-based, exergame	To evaluate how well children understand and apply the dietary energy density principle *Knowledge (P), maintenance of knowledge (S), food intake (S), PA (S), sedentary behavior (S), acceptability (O), emotions (O)*	9–12 years, Germany (82)	*RCT:* IG played game, CG received a paper-pencil brochure about healthy nutrition and PA./*1,5 h in 2 weeks.*	Knowledge scores increased for IG. No behavioral changes were observed for IG or CG, but children liked the game.	Some ^a^ P(1/1) ^b^ S(1/4) ^b^ *Short-term ^c^*
Maes et al. (2011) *Program that consists of a validated Food Frequency Questionnaire, a food composition database, and decision tree to generate tailored advice.*	Web-based	To investigate its feasibility and impact *Nutrient intake (fiber, vitamin C, Ca, Fe, Fat) (P) feasibility (O), acceptability (O)*	12–17 years, from 7 European cities (558)	*QES:* IG used program at computer. CG received paper-pencil standard advice./*1 month*	Intervention was feasible and generally well appreciated. After 1 month, CG reported increased fat intake, which stayed stable for IG. Trend remained after 3 months.	Some ^a^ P(1/5) ^b^ *Medium-term ^c^*
Majumdar et al. (2013) *Game in which players save creatures by helping them to adopt a healthier lifestyle.*	Web-based	To evaluate efficacy in promoting energy balance-related behaviors and decreasing snack intake *Food intake (P), sedentary behavior (P), PA behavior (P)*	11–13 years from low-income schools, US (590)	*QES:* IG played Creature-101 and CG played Whyville*/4 h in 1 month*	IG decreased frequency and number of unhealthy snacks and SSBs consumed. No changes observed in the other behaviors.	Some ^a^ *P*(1/3) ^b^ *Short-term* ^c^
Fraticelli et al. (2016), Marchetti et al. (2015) *Game in which players must find their way back from planet GNAM to earth by dodging obstacles, by eating healthy.*	Web-based	To design, develop, and preliminary evaluate its effect improving knowledge and increasing consumption of healthy foods *Knowledge (O), food intake (O), engagement (O)*	14–18 years, Italy (83)	*E:* Interdisciplinary team designed and developed game. Children played game and completed questionnaire./*7 h in 1 week*	IG showed increased knowledge of healthy food and some changes towards more healthy food choices. Game was easy-to-use and interesting.	N.a.
		To test the improvement of knowledge and to analyze participants' enjoyment *Knowledge (P), engagement (O)*	17–21 years, Italy (65)	*QES:* IG played game, CG played Angry Birds Halloween*/3 sessions in 2 weeks*	IG improved dietary knowledge. Levels of fun experienced did not differ between IG and CG.	Yes *P*(1/1) ^b^ *Short-term* ^c^
Caon et al. (2022), Martin et al. (2020) *Interdisciplinary system consisting of wearable sensors, mobile phones with apps, and multimedia diaries. Incorporates feedback, social connectivity, and engagement.*	Multiple technologies	To describe its co-designing process and feasibility *Feasibility (O), usability (O)*	13–16 years, UK, Italy, and Spain (74)	*E:* 3 iterations evaluated in focus group discussions and questionnaires to inform further development./*60-90 min (focus groups)*	Opportunities for refinement: (1) customizable interface, (2) age-appropriate language, (3) tutorials on how to use the app, (4) a clear end-goal, (5) a reward system, (6) variation in gamification, and (7) social support.	N.a.
		To describe implementation procedures, assess effectiveness, and evaluate usability and engagement *Dietary practices (F, V, SSB, snacks, fast food, breakfast) (P), engagement (O), feasibility (O)*	13–16 years, UK, Italy, and Spain (357)	*QES:* IG is provided with PEGASO system. CG received no intervention./*6 months*.	Higher engagement was associated with improved outcomes. During first weeks overall usability was high, but declined thereafter	Some ^a^ P(2/4) ^b^ *Long-term ^c^*
Mauriello et al. (2010) *Program that addresses recommended health guidelines, by providing tailored feedback.*	Web-based	To evaluate its impact on energy balance behaviors *Food intake (FV) (P), PA (P), sedentary behavior (P), stage of change (S), anthropometrics (S)*	14–17 years, US (1800)	*RCT:* IG received intervention sessions. No-intervention CG./*1.5 h in 2 months.*	Program initiated behavior change across all behaviors and helped adolescents to maintain healthy behaviors. Co-variation of behavior change occurred.	Some ^a^ P(1/3) ^b^ S(0/2) ^b^ *Long-term* ^c^
Merino-Godoy et al. (2022) *App to provide tips related to health behaviors via brief messages, also contains extra information, a game, a forum, and rewards.*	App-based	To analyze its effects on healthy habits *Food intake (P), perception (P)*	8–16 years, Spain (89)	*QES:* IG: app use together with trained person. CG: class-based intervention/*21 h in 4 months*.	No changes in habits of IG observed, but increase was found in the feeling of being fit and healthy.	No P(0/2) ^b^ *Short-term ^c^*
Nollen et al. (2014) *App including goal setting, planning, prompting, self-monitoring, feedback, and reinforcing features, that prompts to set 2 daily goals and action plans.*	App-based	To test the feasibility and potential efficacy *Anthropometry (P), food intake (FV & SSBs) (P), screen time (P), device usability (O)*	9–14 years, economically disadvantaged girls, US (51)	*RCT:* IG used app. CG received paper-pencil manuals with screenshots from modules./*12 weeks*.	No effects were observed on any outcome, but trends towards increased FVs and decreased SSBs were found.	No P(0/4) ^b^ *Medium-term ^c^*
Pedersen et al. (2016) *Two-way text messaging program, which prompts users to set weekly goals and sends a daily report.*	Text message-based	To investigate its impact on FV intake *Food intake (P), self-efficacy (P), outcome expectations (P), engagement (O)*	11–16 years, Denmark (1488)	*RCT:* 4 arms: 2 IGs set daily goals on FV. 1 IG receives an extra nutrition education session. No-intervention CG./*11 weeks.*	No effects were observed on FV in IG, but effects of level of intervention engagement on IG's outcomes were found.	No P(0/3) ^b^ *Short-term ^c^*
Ragelienė et al. (2022) *App in which users can record FV intake, play 2 games to promote healthy food preferences, and have social interaction.*	App-based	To evaluate experiences and evaluate its efficacy in improving dietary outcomes *Food intake (P), preference (P), self-efficacy (P), knowledge (P), usability (O), acceptability (O)*	9–13 years, Denmark (118)	*QES:* IG used app as much as possible during first 2 weeks. No-intervention CG./*3 months*	IG experienced app relatively positive and found app easy-to-use. IG reported increase in FV preference and F intake. No effects for other outcomes.	Some ^a^ P(1/4) ^b^ *Medium-term ^c^*
Rosi et al. (2015) *Program combining videogames, teaching manuals, and a character to guide children through the program.*	Computer-based	To evaluate its effect on improving eating habits *Intake (O)*	8–10 years old, Italy (76)	*E:* IG completed assessments after gameplay. No CG./ *3 months.*	FV consumption and daily dietary total antioxidant capacity increased after the intervention.	N.a.
Ruggiero et al. (2020) *Educational exergame. Contains knowledge questions, movement activities, and educational messages related to healthy eating and PA.*	Computer-based, exergame	To describe the development, and initial feasibility, acceptability, and preliminary outcomes *Knowledge (O), intention (O), acceptability (O)*	7–13 years, US (75)	*E:* 2 iteration phases: (1) examined individual + team play, (2) only team play. No CG/*Phase 1: 4 sessions over 2 weeks. Phase 2: 2 sessions over 2 weeks.*	Youth increased (in-game) knowledge, behavioral intentions to eat more FVs, and PA. Game was reported to be fun.	N.a.
Schneider et al. (2012) *Virtual pet game. By making healthy food choices, the pet becomes healthier and can win more games.*	Web-based	To evaluate its acceptability *Knowledge (O), attitude (O), self-efficacy (O), acceptability (O)*	11 years, US (97)	*E:* IG played game during class. No CG./*4 h in 1 week*	IG increased diet self-efficacy and attitudes towards healthy foods. No change in nutritional knowledge. Game was highly acceptable.	N.a.
Sharma et al. (2015) *Educational game in which children make simulated healthy food choices and should stay physically active.*	Web-based	To evaluate its feasibility, acceptability, and effects *Food intake (P), dietary habits (P), PA (P), attitude (P), self-efficacy (P), knowledge (P), process experience (O), usability (O)*	9–11 years, ethnically diverse, US (107)	*RCT:* IG played game. No-intervention CG*/9 h in 6 weeks.*	IG showed decreased consumption of sugar and higher PA/nutrition attitudes. No effects on PA. The game showed high levels of acceptability and usability.	Some ^a^ P(2/6) ^b^ *Short-term ^c^*
Shukri et al. (2017), Shukri et al. (2019) *Intervention including interactive games and an animated presentation designed to support intervention modules.*	Computer-based	To evaluate its effects on attitude, intention, and dietary habits *Attitudes (P), intentions (P), food intake (P)*	10–11 years, socially disadvantaged, Malaysia (51)	*QES:* IG used intervention program and traditional methods. CG received PowerPoint presentation/*3 weeks (1 d per week)*	IG showed positive changes in attitude towards some unhealthy foods, and in consumption of V, fast food, SSB, and sweet foods.	No P(0/3) ^b^ *Medium-term ^c^*
		To evaluate its effects on knowledge, attitudes, intentions, and dietary intake *Knowledge (P), attitudes (P), intentions (P), food intake (P)*	10–11 years, socially disadvantaged, Malaysia (201)	*QES:* IG used intervention and methods. CG received PowerPoint presentation/*3 weeks (1 d per week)*.	No differences in knowledge and intentions were found. Positive effects were found on attitude towards unhealthy foods, consumption of V and unhealthy foods. Effects did not sustain.	Some ^a^ P(2/4) ^b^ *Long-term ^c^*
Silva et al. (2015) *Text messaging program. Algorithm evaluates FV, PA and screen time reports on set goals met. Program automatically sends tailored feedback messages back.*	Text message-based	To evaluate its effectiveness in increasing V consumption and PA and decreasing screen time *Food intake (P), PA (P), sedentary behavior (P), program experience (O)*	8–10 years, Portugal (139)	*RCT:* IG + CG attended 2 educational sessions. IG monitored health behaviors via SMS. No-intervention CG./*8 weeks.*	IG increased FV consumption. No effect on PA and screen time was observed. IG was satisfied with intervention.	Some ^a^ P(1/3) ^b^ *Short-term ^c^*
de Sousa et al. (2020), Sousa et al. (2020) *Program consisting of app (with educational resources, social support, self-monitoring, interactive training modules, and motivational features) and face-to-face sessions on healthy behaviors.*	App-based	To evaluate its effectiveness and analyze the success-predictors *Health responsibility (P), PA (P), dietary habits (P), positive life perspective (P), interpersonal relations (P), stress management (P), spiritual health (P), global lifestyle (P), predictors of effectiveness (O)*	12–16 years, Portugal, (24)	*QES:* IG used app in addition to a structured school-based intervention CG only followed school-based intervention*/6 months.*	IG showed effect on nutrition, positive life perspective, and global lifestyle scores. Older adolescents tended to show an increase in stress management rates.	Some ^a^ P(4/8) ^b^ *Long-term ^c^*
		To assess its acceptance *Acceptability (usefulness, ease-of-use, attitude, intention) (O)*	12–16 years, Portugal (47)	*E:* Questionnaire + online discussion forum*/Not specified.*	Program was acceptable for all 4 acceptability factors assessed.	N.a.
Cullen et al. (2016), DeSmet et al. (2017), Thompson et al. (2015) *Game in which players (squires) must acquire and overcome knowledge, skills, and overcome challenges related to FVs, to ultimately become a knight.*	Web-based	To evaluate the short- and long-term effects of goal setting enhanced with implementation intentions on child FV intake *Food intake (FV) (P), food intake (F, V) (S), program usage (O), program satisfaction (O)*	8–11 years, plus parents, US (400)	RCT: 4 arms: None (CG), Action, Coping, Action and Coping. IG is supported by parent-components, e.g. digital newsletter and access parent- website*/4 h in 3 months.*	Increases in FV intake were observed in Action group immediate post-test and at follow-up. In Coping group, increases were found at immediate post-test. No other effects were found.	Some ^a^ P(1/1) ^b^ S(1/2) ^b^ *Medium-term* ^c^
		To assess meal-specific changes in FV intake *Food intake (FV: breakfast, lunch, snack, & dinner) (P)*	8–11 years, plus parents, US (400)	RCT: 4 arms: None (CG), Action, Coping, Action and Coping. IG is supported by parent-components, e.g. digital newsletter and access parent- website*/4 h in 3 months.*	Action and Coping group participants reported higher vegetable intake at dinner. Increases over time for fruit was observed for breakfast, lunch, and snacks at follow-up.	Some ^a^ P(1/4) ^b^ *Medium-term* ^b^
		To assess if asking behavior to improve home FV availability predicts FV home availability and FV intake *FV asking behavior (P), home FV availability (P), food intake (FV) (P)*	8–11 years, plus parents, US (400)	RCT: 4 arms: None (CG), Action, Coping, Action and Coping. IG is supported by parent-components, e.g. digital newsletter and access parent- website*/4 h in 3 months.*	Baseline asking behaviors to improve home FV availability predicted baseline FV availability. IG increased home availability child asking behaviors, but the latter did not predict home availability and intake.	Some ^a^ P(4/6) ^b^ *Medium-term ^c^*
Champion et al. (2020), Thornton et al. (2021) *App in which adolescents track and set goals for healthy lifestyle behaviors, using in-app rewards, and notifications.*	App-based	To summarize the collaborative design and user testing *Acceptability (O), applicability (O)*	12–16 years, Australia (50)	*E:* User tests with students (focus groups) and teachers (web-survey)/*N.a.*	App was perceived acceptable and relevant. Areas for improvement include: length, age-appropriateness, character backstories, links to information, and implementation feasibility.	N.a.
		To describe its development, usability, and acceptability *Usability (O), acceptability (O)*	12–16 years, Australia (232)	*E:* App was iteratively developed by involving experts and adolescents in 3 phases/*N.a.*	App was well accepted and usable. Areas for improvement include: interactive features, display of recorded behaviors.	N.a.
Vepsäläinen et al. (2022) *App in which users complete different tasks, individually (minigames) and in a group (adult-led) to familiarize with different FVs. Also contains a taste bank.*	App-based	To describe the development and evaluate its effectiveness. *Acceptance (FV) (P), relative acceptance (P)*	3–6 years, Poland and Finland (221)	*QES:* IG used app, care professionals recorded tasks completed and FVs introduced. CG continued normal routines/*1 or 2 times per week for 4 weeks.*	IG had higher FV acceptance score and relative FV acceptance scores compared to CG.	Yes P(2/2) ^b^ *Short-term ^c^*
Whittemore et al. (2013) *Program consisting of goal setting, self-monitoring, health coaching, and social networking features.*	Web-based	To compare the effect of two programs on health behaviors *Anthropometrics (P), sedentary behavior (S), PA (S), food intake (S) self-efficacy (S), acceptability (O), program usability (O)*	14–16 years, diverse ethnicities, US (384)	*RCT:* 2 arms: 1 received intervention only, other received intervention + coping skills training/*8 lessons*	Improvements in sedentary behavior, PA, healthy eating, and self-efficacy were found for IG and CG. Effects did not sustain over time. Participants were satisfied with program.	Some ^a^ P(0/1) ^b^ S(2/4) ^b^ *Long-term ^c^*

IG – Intervention group; CG – Control group; RCT – Randomized Controlled Trial; QES – Quasi-Experimental Study; E – Exploratory; F – Fruit; V – Vegetable; FV – Fruit and Vegetable; SSB – Sugar Sweetened Beverage; PA – Physical Activity; US – United States; UK – United Kingdom; P – Primary objective(s); S – Secondary objective(s); O – Other outcome(s).

^a^ Studies were labeled as having ‘some favorable outcomes’ when significant improvements (*p* < 0.05) in favor of DBDI were found for some primary or secondary outcomes, but not all, or when effects did not maintain at follow-up assessments (117).

^b^ Proportion of effects on primary (P) and secondary (S) outcomes. Intervention was considered as being favorable for S or P when DBDI significantly (*p* < 0.05) changed at least >50% of outcome determinant within category of outcomes (i.e. specific food products within category *food intake*).

^c^ Duration of outcomes in favor of DBDI was considered: (1) short-term: effects assessed ≤1 month post-intervention, (2) medium-term: effects assessed ≤3 months post-intervention, (3) long-term: effects assessed ≥6 months post-intervention.

In total, 51 articles were included describing 41 DBDIs from 17 countries on 5 continents, including around 12,000 participants (ranging from *n* = 34 to *n* = 1800 per study). About half of the studies (*n* = 22/51, 43%) had sample sizes under 100 participants (Ahn et al., 2016; Alblas et al., 2020; Brown et al., 2020; Byrne et al., 2012; Champion et al., 2020; Chen et al., 2011; de Sousa et al., 2020; Espinosa-Curiel et al., 2020; Espinosa-Curiel et al., 2022; Farrow et al., 2019; Fraticelli et al., 2016; Froome et al., 2020; Mack et al., 2020; Marchetti et al., 2015; Martin et al., 2020; Merino-Godoy et al., 2022; Nollen et al., 2014; Rosi et al., 2015; Ruggiero et al., 2020; Schneider et al., 2012; Shukri et al., 2017; Wang et al., 2015). Most studies were experimental (*n* = 40/51, 78%) (Ahn et al., 2016; Alblas et al., 2020; Baranowski et al., 2011; Baranowski et al., 2019; Bell et al., 2018; Benavides et al., 2021; Byrne et al., 2012; Caon et al., 2022; Chamberland et al., 2017; Chang et al., 2022; Chen et al., 2011; Cullen et al., 2013; Cullen et al., 2016; De Cock et al., 2018; DeSmet et al., 2017; dos Santos Chagas et al., 2020; Espinosa-Curiel et al., 2022; Ezendam et al., 2012; Farrow et al., 2019; Folkvord et al., 2021; Fraticelli et al., 2016; Froome et al., 2020; Hermans et al., 2018; Kato-Lin et al., 2020; Mack et al., 2020; Maes et al., 2011; Majumdar et al., 2013; Mauriello et al., 2010; Merino-Godoy et al., 2022; Nollen et al., 2014; Pedersen et al., 2016; Ragelienė et al., 2022; Sharma et al., 2015; Shukri et al., 2017; Shukri et al., 2019; Sousa et al., 2020; Thompson et al., 2015; Vepsäläinen et al., 2022; Whittemore et al., 2013), with a study duration ranging from 10 min to 14 months; particularly RCT (*n* = 23/51, 45%) (Ahn et al., 2016; Baranowski et al., 2011; Baranowski et al., 2019; Byrne et al., 2012; Chamberland et al., 2017; Chen et al., 2011; Cullen et al., 2013; Cullen et al., 2016; DeSmet et al., 2017; dos Santos Chagas et al., 2020; Ezendam et al., 2012; Farrow et al., 2019; Folkvord et al., 2021; Froome et al., 2020; Kato-Lin et al., 2020; Mack et al., 2020; Mauriello et al., 2010; Nollen et al., 2014; Pedersen et al., 2016; Sharma et al., 2015; Silva et al., 2015; Thompson et al., 2015; Whittemore et al., 2013), followed by QES (*n* = 17/51, 33%), i.e. non-RCTs (Alblas et al., 2020; Bell et al., 2018; Benavides et al., 2021; Caon et al., 2022; Chang et al., 2022; De Cock et al., 2018; Espinosa-Curiel et al., 2022; Fraticelli et al., 2016; Hermans et al., 2018; Maes et al., 2011; Majumdar et al., 2013; Merino-Godoy et al., 2022; Ragelienė et al., 2022; Shukri et al., 2017; Shukri et al., 2019; Sousa et al., 2020; Vepsäläinen et al., 2022). The remaining studies (*n* = 11/51, 20%) were classified as exploratory (Brown et al., 2020; Champion et al., 2020; de Sousa et al., 2020; Espinosa-Curiel et al., 2020; Marchetti et al., 2015; Martin et al., 2020; Rosi et al., 2015; Ruggiero et al., 2020; Schneider et al., 2012; Thornton et al., 2021; Wang et al., 2015), including qualitative studies, mixed methods studies, or studies without control group.

### Summary of included DBDIs

#### Technological delivery modes

Among 41 DBDIs, 5 groups of technological delivery modes were distinguished: app-based (*n* = 15/41, 37%) (Bell et al., 2018; Benavides et al., 2021; Brown et al., 2020; Byrne et al., 2012; Champion et al., 2020; De Cock et al., 2018; de Sousa et al., 2020; dos Santos Chagas et al., 2020; Farrow et al., 2019; Folkvord et al., 2021; Froome et al., 2020; Kato-Lin et al., 2020; Martin et al., 2020; Merino-Godoy et al., 2022; Nollen et al., 2014; Ragelienė et al., 2022; Sousa et al., 2020; Thornton et al., 2021; Vepsäläinen et al., 2022), web-based (*n* = 12/41, 29%) (Chamberland et al., 2017; Chen et al., 2011; Cullen et al., 2013; Cullen et al., 2016; DeSmet et al., 2017; Ezendam et al., 2012; Fraticelli et al., 2016; Maes et al., 2011; Majumdar et al., 2013; Marchetti et al., 2015; Mauriello et al., 2010; Schneider et al., 2012; Sharma et al., 2015; Thompson et al., 2015; Whittemore et al., 2013), computer-based (*n* = 11/41, 27%) (Ahn et al., 2016; Alblas et al., 2020; Baranowski et al., 2011; Baranowski et al., 2019; Chang et al., 2022; Espinosa-Curiel et al., 2020; Espinosa-Curiel et al., 2022; Hermans et al., 2018; Mack et al., 2020; Rosi et al., 2015; Ruggiero et al., 2020; Shukri et al., 2017; Shukri et al., 2019; Wang et al., 2015), text-message-based (*n* = 2/41, 5%) (Pedersen et al., 2016; Silva et al., 2015), or a combination of technologies (*n* = 1/41, 2%) (Caon et al., 2022; Martin et al., 2020). To illustrate, Rageliene et al (Ragelienė et al., 2022) made use of an app-based approach that consisted of a food diary, games, and the possibility to interact with peers. Martin et al. (2020) and Caon et al. (2022) combined wearable sensors, mobile phone apps, and games, as part of an international intervention for improved health outcomes (Caon et al., 2022; Martin et al., 2020). Of the 41 included DBDIs, 12 (29%) were supported by non-technological intervention aspects (Bell et al., 2018; Champion et al., 2020; de Sousa et al., 2020; Mack et al., 2020; Maes et al., 2011; Merino-Godoy et al., 2022; Pedersen et al., 2016; Ruggiero et al., 2020; Shukri et al., 2017; Shukri et al., 2019; Silva et al., 2015; Sousa et al., 2020; Thornton et al., 2021; Vepsäläinen et al., 2022; Whittemore et al., 2013). Bell et al. (2018), for example, combined an app-based game with a special intervention curriculum for 1 h a week, including a family home activity (Bell et al., 2018). Other 29 DBDIs (71%) functioned as a primary (stand-alone) intervention (Ahn et al., 2016; Alblas et al., 2020; Benavides et al., 2021; Brown et al., 2020; Byrne et al., 2012; Caon et al., 2022; Chang et al., 2022; Chen et al., 2011; Cullen et al., 2013; Cullen et al., 2016; De Cock et al., 2018; DeSmet et al., 2017; dos Santos Chagas et al., 2020; Espinosa-Curiel et al., 2020; Espinosa-Curiel et al., 2022; Ezendam et al., 2012; Farrow et al., 2019; Folkvord et al., 2021; Fraticelli et al., 2016; Froome et al., 2020; Hermans et al., 2018; Kato-Lin et al., 2020; Majumdar et al., 2013; Marchetti et al., 2015; Martin et al., 2020; Mauriello et al., 2010; Nollen et al., 2014; Ragelienė et al., 2022; Rosi et al., 2015; Schneider et al., 2012; Sharma et al., 2015; Thompson et al., 2015). A commonly applied DBDI design feature was gamification (*n* = 27/41, 66%) (Ahn et al., 2016; Alblas et al., 2020; Baranowski et al., 2011; Baranowski et al., 2019; Bell et al., 2018; Brown et al., 2020; Byrne et al., 2012; Chang et al., 2022; Cullen et al., 2016; De Cock et al., 2018; DeSmet et al., 2017; dos Santos Chagas et al., 2020; Espinosa-Curiel et al., 2020; Espinosa-Curiel et al., 2022; Farrow et al., 2019; Folkvord et al., 2021; Fraticelli et al., 2016; Froome et al., 2020; Hermans et al., 2018; Kato-Lin et al., 2020; Mack et al., 2020; Majumdar et al., 2013; Marchetti et al., 2015; Merino-Godoy et al., 2022; Ragelienė et al., 2022; Rosi et al., 2015; Ruggiero et al., 2020; Schneider et al., 2012; Sharma et al., 2015; Shukri et al., 2017; Shukri et al., 2019; Vepsäläinen et al., 2022; Wang et al., 2015), for example through care for virtual pets (Ahn et al., 2016; Byrne et al., 2012; Chang et al., 2022; Schneider et al., 2012), robots (Brown et al., 2020; Froome et al., 2020), or gardens (Bell et al., 2018), action-adventure gaming elements (Baranowski et al., 2011; Baranowski et al., 2019; Chang et al., 2022; Cullen et al., 2016; De Cock et al., 2018; DeSmet et al., 2017; Espinosa-Curiel et al., 2022; Folkvord et al., 2021; Fraticelli et al., 2016; Kato-Lin et al., 2020; Mack et al., 2020; Majumdar et al., 2013; Marchetti et al., 2015; Sharma et al., 2015; Thompson et al., 2015; Wang et al., 2015), combining exercise with gaming (i.e. *exergaming*) (Espinosa-Curiel et al., 2022; Hermans et al., 2018; Mack et al., 2020; Ruggiero et al., 2020), educational card games (dos Santos Chagas et al., 2020), or teamplay (Vepsäläinen et al., 2022). To illustrate, in the game by Froome et al. (2020) and Brown et al. (2020) players help robots in making healthy food choices with virtual nutrition scientists (Brown et al., 2020; Froome et al., 2020). Ahn et al. (Ahn et al., 2016), on the other hand, used a virtual pet to promote fruit and vegetable consumption by seeing, hearing, and feeling how fruit and vegetables affect their dog’s health. Ruggiero et al. (2020) let users physically engage in knowledge questions, fulfill sports activities, and receive motivational messages related to physical activity and healthy eating (Ruggiero et al., 2020).

#### Design and development

Most DBDIs were developed and evaluated in high income countries (*n* = 35/41, 86%): United States (*n* = 14/41, 37%) (Ahn et al., 2016; Baranowski et al., 2011; Baranowski et al., 2019; Bell et al., 2018; Byrne et al., 2012; Chen et al., 2011; Cullen et al., 2013; Cullen et al., 2016; DeSmet et al., 2017; Majumdar et al., 2013; Mauriello et al., 2010; Nollen et al., 2014; Ruggiero et al., 2020; Schneider et al., 2012; Sharma et al., 2015; Thompson et al., 2015; Wang et al., 2015; Whittemore et al., 2013), the Netherlands (*n* = 4/41, 10%) (Alblas et al., 2020; Ezendam et al., 2012; Folkvord et al., 2021; Hermans et al., 2018), Denmark (*n* = 2/41 5%) (Pedersen et al., 2016; Ragelienė et al., 2022), Canada (*n* = 2/41, 5%) (Brown et al., 2020; Chamberland et al., 2017; Froome et al., 2020), Belgium (*n* = 1/41, 2%) (De Cock et al., 2018), Italy (*n* = 2/41, 5%) (Fraticelli et al., 2016; Marchetti et al., 2015; Rosi et al., 2015), Spain (*n* = 2/41, 5%) (Benavides et al., 2021; Merino-Godoy et al., 2022), Portugal (*n* = 2/41, 5%) (de Sousa et al., 2020; Silva et al., 2015; Sousa et al., 2020), Australia (*n* = 1/41, 2%) (Champion et al., 2020; Thornton et al., 2021), Germany (*n* = 1/41, 2%) (Mack et al., 2020), Finland (*n* = 1/41, 2%) (Vepsäläinen et al., 2022), United Kingdom (UK) (*n* = 1/41, 2%) (Farrow et al., 2019), or a multi-country study in several high income countries (*n* = 2/41, 2%) (Caon et al., 2022; Maes et al., 2011; Martin et al., 2020). The remaining 7 studies (14%) were conducted in low- and middle-income countries (Chang et al., 2022; dos Santos Chagas et al., 2020; Espinosa-Curiel et al., 2020; Espinosa-Curiel et al., 2022; Kato-Lin et al., 2020; Shukri et al., 2017; Shukri et al., 2019): Mexico (*n* = 2/51, 4%) (Espinosa-Curiel et al., 2020; 2022), India, (*n* = 1/51, 2%) (Kato-Lin et al., 2020), Brazil (*n* = 1/51, 2%) (dos Santos Chagas et al., 2020), Malaysia (*n* = 2/51, 4%) (Shukri et al., 2017; Shukri et al., 2019), and Taiwan (*n* = 1/51, 2%) (Chang et al., 2022). For DBDIs in our sample, 59% (*n* = 24) involved stakeholder in the developmental process (Baranowski et al., 2011; Baranowski et al., 2019; Bell et al., 2018; Benavides et al., 2021; Brown et al., 2020; Caon et al., 2022; Champion et al., 2020; Chang et al., 2022; Cullen et al., 2013; Cullen et al., 2016; De Cock et al., 2018; de Sousa et al., 2020; DeSmet et al., 2017; dos Santos Chagas et al., 2020; Espinosa-Curiel et al., 2020; Espinosa-Curiel et al., 2022; Folkvord et al., 2021; Fraticelli et al., 2016; Froome et al., 2020; Hermans et al., 2018; Marchetti et al., 2015; Martin et al., 2020; Merino-Godoy et al., 2022; Nollen et al., 2014; Ragelienė et al., 2022; Ruggiero et al., 2020; Sharma et al., 2015; Shukri et al., 2017; Shukri et al., 2019; Sousa et al., 2020; Thompson et al., 2015; Thornton et al., 2021; Vepsäläinen et al., 2022; Wang et al., 2015). Most frequently involved stakeholders include end-users (i.e. children) (*n* = 14/41, 34%) (Baranowski et al., 2011; Baranowski et al., 2019; Benavides et al., 2021; Caon et al., 2022; Champion et al., 2020; Chang et al., 2022; Cullen et al., 2013; Cullen et al., 2016; De Cock et al., 2018; DeSmet et al., 2017; dos Santos Chagas et al., 2020; Espinosa-Curiel et al., 2020; Martin et al., 2020; Merino-Godoy et al., 2022; Nollen et al., 2014; Sharma et al., 2015; Thompson et al., 2015; Thornton et al., 2021; Vepsäläinen et al., 2022; Wang et al., 2015), nutritionists (*n* = 11/41, 27%) (Bell et al., 2018; Benavides et al., 2021; Brown et al., 2020; Caon et al., 2022; de Sousa et al., 2020; Espinosa-Curiel et al., 2020; Espinosa-Curiel et al., 2022; Fraticelli et al., 2016; Froome et al., 2020; Hermans et al., 2018; Marchetti et al., 2015; Martin et al., 2020; Ruggiero et al., 2020; Shukri et al., 2017; Shukri et al., 2019; Sousa et al., 2020), and psychologists (*n* = 7/41, 17%) (Bell et al., 2018; de Sousa et al., 2020; Espinosa-Curiel et al., 2020; Espinosa-Curiel et al., 2022; Fraticelli et al., 2016; Marchetti et al., 2015; Ruggiero et al., 2020; Shukri et al., 2017; Shukri et al., 2019; Sousa et al., 2020). Only 2 (5%) explicitly mentioned using co-design methodologies to meet user needs (Caon et al., 2022; Champion et al., 2020; Martin et al., 2020; Thornton et al., 2021).

##### Target populations

Among DBDIs developed for use in high-income countries (*n* = 35/41, 86%), 7 (20%) specifically targeted minority populations of children, including ethnically diverse children (*n* = 5/35, 14%) (Bell et al., 2018; Chen et al., 2011; Sharma et al., 2015; Wang et al., 2015; Whittemore et al., 2013), children from low-income families/schools (*n* = 2/35, 6%) (Majumdar et al., 2013; Nollen et al., 2014), and girls (*n* = 1/35, 3%) (Nollen et al., 2014). Among DBDIs designed for low- and middle-income countries (*n* = 6, 15%), only 1 targeted a minority population, i.e. socially disadvantaged children (Shukri et al., 2017; Shukri et al., 2019). Moreover, when classifying DBDIs into target age categories, almost half (*n* = 19/41, 47%) aimed at children between 11-14 years (Ahn et al., 2016; Alblas et al., 2020; Baranowski et al., 2011; Baranowski et al., 2019; Bell et al., 2018; Benavides et al., 2021; Brown et al., 2020; Byrne et al., 2012; Caon et al., 2022; Chamberland et al., 2017; Champion et al., 2020; Chen et al., 2011; Cullen et al., 2013; Cullen et al., 2016; De Cock et al., 2018; de Sousa et al., 2020; DeSmet et al., 2017; dos Santos Chagas et al., 2020; Espinosa-Curiel et al., 2022; Ezendam et al., 2012; Folkvord et al., 2021; Hermans et al., 2018; Kato-Lin et al., 2020; Mack et al., 2020; Maes et al., 2011; Majumdar et al., 2013; Marchetti et al., 2015; Martin et al., 2020; Mauriello et al., 2010; Merino-Godoy et al., 2022; Nollen et al., 2014; Pedersen et al., 2016; Ragelienė et al., 2022; Ruggiero et al., 2020; Schneider et al., 2012; Sharma et al., 2015; Sousa et al., 2020; Thompson et al., 2015; Thornton et al., 2021; Whittemore et al., 2013), followed by children aged 7–10 years (*n* = 11/41, 27%) (Ahn et al., 2016; Baranowski et al., 2011; Baranowski et al., 2019; Bell et al., 2018; Brown et al., 2020; Cullen et al., 2016; DeSmet et al., 2017; Espinosa-Curiel et al., 2020; Espinosa-Curiel et al., 2022; Folkvord et al., 2021; Froome et al., 2020; Hermans et al., 2018; Kato-Lin et al., 2020; Mack et al., 2020; Merino-Godoy et al., 2022; Nollen et al., 2014; Ragelienė et al., 2022; Rosi et al., 2015; Ruggiero et al., 2020; Sharma et al., 2015; Silva et al., 2015; Thompson et al., 2015), and adolescents aged 15–18 years (*n* = 8/41, 20%) (Caon et al., 2022; Champion et al., 2020; Chen et al., 2011; Cullen et al., 2013; De Cock et al., 2018; de Sousa et al., 2020; dos Santos Chagas et al., 2020; Fraticelli et al., 2016; Maes et al., 2011; Marchetti et al., 2015; Martin et al., 2020; Mauriello et al., 2010; Merino-Godoy et al., 2022; Pedersen et al., 2016; Sousa et al., 2020; Thornton et al., 2021; Whittemore et al., 2013). The minority of the DBDIs focused on children of 3–6 years (*n* = 3/41, 7%) (Chang et al., 2022; Farrow et al., 2019; Vepsäläinen et al., 2022) or adolescents of 19–21 years old (*n* = 2, 5%) (dos Santos Chagas et al., 2020; Fraticelli et al., 2016).

### Behavioral theory

#### Behavior change theories and frameworks

Among included DBDIs, 32 (78%) used behavioral theory or theoretical frameworks as the foundation for DBDI development (Ahn et al., 2016; Alblas et al., 2020; Baranowski et al., 2011; Baranowski et al., 2019; Bell et al., 2018; Benavides et al., 2021; Brown et al., 2020; Byrne et al., 2012; Caon et al., 2022; Chamberland et al., 2017; Champion et al., 2020; Chen et al., 2011; Cullen et al., 2013; Cullen et al., 2016; De Cock et al., 2018; DeSmet et al., 2017; dos Santos Chagas et al., 2020; Espinosa-Curiel et al., 2020; Espinosa-Curiel et al., 2022; Ezendam et al., 2012; Folkvord et al., 2021; Fraticelli et al., 2016; Froome et al., 2020; Hermans et al., 2018; Kato-Lin et al., 2020; Majumdar et al., 2013; Marchetti et al., 2015; Martin et al., 2020; Mauriello et al., 2010; Merino-Godoy et al., 2022; Nollen et al., 2014; Ragelienė et al., 2022; Ruggiero et al., 2020; Sharma et al., 2015; Silva et al., 2015; Thompson et al., 2015; Thornton et al., 2021; Vepsäläinen et al., 2022; Wang et al., 2015; Whittemore et al., 2013). Specifically, 20 different theories were referred to, with social cognitive theory (*n* = 20, 49%) (Ahn et al., 2016; Alblas et al., 2020; Baranowski et al., 2011; Baranowski et al., 2019; Byrne et al., 2012; Champion et al., 2020; Cullen et al., 2013; dos Santos Chagas et al., 2020; Espinosa-Curiel et al., 2020; Fraticelli et al., 2016; Kato-Lin et al., 2020; Majumdar et al., 2013; Marchetti et al., 2015; Ragelienė et al., 2022; Ruggiero et al., 2020; Silva et al., 2015; Thornton et al., 2021; Vepsäläinen et al., 2022; Wang et al., 2015), BCTTv1 (*n* = 16/41, 39%) (Bell et al., 2018; Benavides et al., 2021; Brown et al., 2020; Caon et al., 2022; Chamberland et al., 2017; Champion et al., 2020; Cullen et al., 2013; Cullen et al., 2016; De Cock et al., 2018; DeSmet et al., 2017; Espinosa-Curiel et al., 2020; Espinosa-Curiel et al., 2022; Fraticelli et al., 2016; Froome et al., 2020; Hermans et al., 2018; Majumdar et al., 2013; Marchetti et al., 2015; Martin et al., 2020; Merino-Godoy et al., 2022; Thompson et al., 2015; Thornton et al., 2021), self-determination theory (*n* = 6/41, 15%) (Baranowski et al., 2011; Baranowski et al., 2019; Bell et al., 2018; Champion et al., 2020; Cullen et al., 2016; DeSmet et al., 2017; Fraticelli et al., 2016; Majumdar et al., 2013; Marchetti et al., 2015; Thompson et al., 2015; Thornton et al., 2021; Wang et al., 2015), theory of planned behavior (*n* = 3/41, 7%) (Ezendam et al., 2012; Ragelienė et al., 2022; Sharma et al., 2015), and transtheoretical model of change (*n* = 3/41, 7%) applied most frequently (Chen et al., 2011; Fraticelli et al., 2016; Marchetti et al., 2015; Mauriello et al., 2010).

#### BCTs

**Figure 3.**
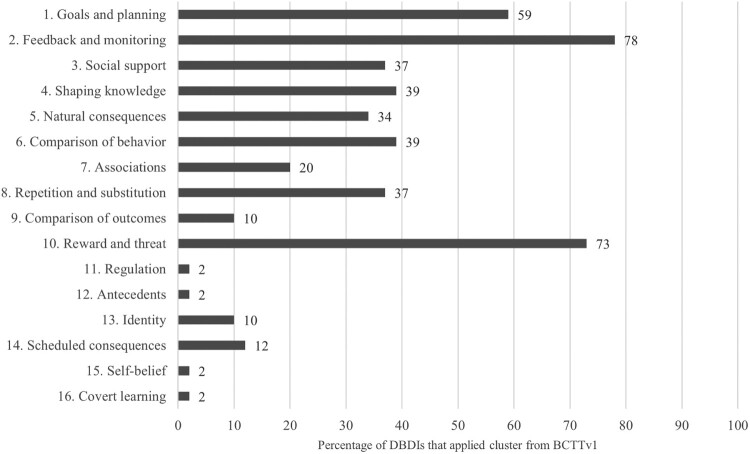
Percentage of digital behavioral dietary interventions (*n* = 41) that incorporated clusters of behavior change techniques from Behavior Change Technique Taxonomy version 1.

Only 16 (39%) of DBDIs explicitly reported incorporating BCTs. For the remaining 25 (61%) DBDIs, BCTs were coded using the BCTTv1 from intervention descriptions to capture implicit incorporation. On average, each DBDI used 6.2 (range 1–21) BCTs. Table 2 details the types, number, and diversity of BCTs included, and specifies which interventions reported on the BCTs they integrated. Figure 3 depicts the percentage of DBDIs applying specific BCT clusters from the BCTTv1 in our sample (*n* = 41).

**Table 2.** Coded behavior change techniques included in digital behavioral dietary interventions using the Behavior Change Techniques Taxonomy v1.

First author (year)	Ahn (2016)	Alblas (2020)	Baranowski (2011; 2019), & Wang (2015)	Bell (2018)	Benavides (2021)	Byrne et al (2012)	Chamberland (2017)	Chang (2022)	Chen (2011)	Cullen (2013)	De Cock (2018)	Dos Santos Chagas (2020)	Espinosa-Curiel (2020)	Espinosa-Curiel (2022)	Ezendam (2012)	Farrow (2019)	Folkvord (2021)	Froome (2020) & Brown (2020)	Hermans (2018)	Kato-Lin (2020)	Mack (2020)	Maes (2011)	Majumdar (2013)	Marchetti (2015) & Fraticelli (2016)	Martin (2020) & Caon (2022)	Mauriello (2010)	Merino-Godoy (2022)	Nollen (2014)	Pedersen (2016)	Rageliene (2022)	Rosi (2015)	Ruggiero (2020)	Schneider (2012)	Sharma (2015)	Shukri (2017, 2019)	Silva (2015)	Sousa (2020) & Lopes de Sousa (2020)	Thompson (2015), Cullen (2013), & Desmet (2017)	Thornton (2021) & Champion (2020)	Vepsäläinen (2022)	Whittemore (2013)	Total
**Behavior change techniques**																																										
1.1 Goal setting (behavior)	✓		✓				✓				✓			✓	✓		✓						✓		✓			✓	✓				✓			✓		✓		✓		**15**
1.2 Problem solving			✓	✓			✓		✓	✓	✓				✓								✓		✓	✓								✓				✓			✓	**13**
1.3 Goal setting (outcome)									✓	✓			✓											✓	✓														✓		✓	**7**
1.4 Action planning			✓						✓	✓					✓								✓					✓										✓				**7**
1.5 Review behavioral goals			✓											✓			✓											✓	✓									✓				**6**
1.6 Discrepancy between current behavior and goal														✓															✓													**2**
1.7 Review outcome goal(s)				✓						✓																																**2**
1.9 Commitment																																						✓				**1**
2.2 Feedback on behavior			✓	✓		✓	✓				✓		✓	✓	✓		✓	✓	✓	✓					✓	✓	✓	✓	✓	✓	✓	✓	✓			✓		✓				**23**
2.3 Self-monitoring of behavior							✓		✓		✓										✓	✓	✓		✓			✓	✓	✓	✓					✓	✓	✓	✓			**15**
2.4 Self-monitoring of outcome(s) of behavior										✓													✓														✓				✓	**4**
2.5 Monitoring of outcome(s) of behavior without feedback														✓																												**1**
2.7 Feedback on outcome(s) of behavior																								✓	✓																✓	**3**
3.1 Social support (unspecified)					✓		✓				✓						✓	✓			✓		✓		✓		✓	✓									✓			✓	✓	**12**
3.2 Social support (practical)					✓										✓																											**2**
3.3 Social support (emotional)				✓																																						**1**
4.1 Instructions on how to perform the behavior			✓		✓				✓	✓			✓	✓	✓		✓	✓						✓	✓		✓						✓			✓		✓		✓		**16**
4.2 Information about antecedents																		✓																								**1**
5.1 Information on health consequences													✓	✓	✓		✓	✓					✓		✓									✓	✓							**9**
5.2 Salience of consequences	✓	✓											✓							✓																						**4**
5.3 Information about social and environmental consequences	✓	✓																					✓		✓							✓				✓						**6**
5.4 Monitoring of emotional consequences														✓																												**1**
6.1 Demonstration of the behavior				✓						✓			✓			✓	✓	✓									✓			✓		✓						✓		✓	✓	**12**
6.2 Social comparison											✓		✓		✓								✓							✓												**5**
6.3 Information about others’ approval					✓																									✓												**2**
7.1 Prompts/cues													✓	✓	✓		✓		✓				✓		✓			✓														**8**
8.1 Behavioral practice/rehearsal				✓					✓			✓	✓	✓					✓	✓										✓	✓		✓	✓	✓		✓			✓		**14**
8.2 Behavioral substitution									✓				✓	✓																												**3**
8.3 Habit formation													✓																													**1**
8.4 Habit reversal													✓																													**1**
8.7 Graded tasks													✓				✓																									**2**
9.1 Credible source																							✓		✓				✓													**3**
9.2 Pros and cons													✓																													**1**
10.1 Material incentive (behavior)		✓																											✓			✓										**3**
10.2 Material reward (behavior)																✓												✓					✓									**3**
10.3 Non-specific reward				✓	✓		✓	✓			✓	✓	✓	✓			✓	✓					✓	✓			✓			✓	✓	✓		✓			✓	✓				**19**
10.4 Social reward			✓										✓						✓																	✓						**4**
10.6 Self-incentive	✓													✓													✓			✓											✓	**5**
10.10 Reward (outcome)				✓																																						**1**
11.2 Reduce negative emotions																																									✓	**1**
12.5 Adding objects to the environment																									✓																	**1**
13.1 Identification of self as role model													✓																													**1**
13.2 Framing/reframing				✓																																						**1**
13.4 Valued self-identify			✓																																			✓				**2**
14.2 Punishment								✓				✓												✓																		**3**
14.3 Remove reward																																	✓									**1**
14.4 Reward approximation													✓											✓																		**2**
14.5 Rewarding completion													✓																													**1**
14.7 Reward incompatible behavior													✓																													**1**
15.4 Self-talk																																						✓				**1**
16.3 Vicarious consequences													✓																													**1**
**Total**	**4**	**3**	**8**	**9**	**5**	**1**	**6**	**2**	**7**	**7**	**7**	**3**	**21**	**13**	**9**	**2**	**10**	**7**	**4**	**3**	**2**	**1**	**12**	**6**	**13**	**2**	**6**	**8**	**7**	**8**	**4**	**5**	**6**	**4**	**2**	**6**	**5**	**12**	**2**	**5**	**8**	

^a^ Studies highlighted in grey explicitly reported behavior change techniques integrated within their interventions.

Most DBDIs incorporated ≥1 BCT from the ‘*Feedback and monitoring’* cluster (*n* = 32/41, 78%). The most used BCT from this cluster was ‘*Feedback on behavior’* (*n* = 22/41, 54%), such as an intervention by Thompson et al. (2015), Cullen et al. (2016), and DeSmet et al. (2017) (Cullen et al., 2016; DeSmet et al., 2017; Thompson et al., 2015), where players receive tailored feedback on meeting fruits and vegetables-related goals. Additionally, the majority of DBDIs applied a BCT from the ‘*Reward and threat’* cluster (*n* = 30, 73%), mostly ‘*Non-specific rewards’* (*n* = 19/41, 46%), such as a wall of fame (de Sousa et al., 2020; Sousa et al., 2020), scoring points (Chang et al., 2022; de Sousa et al., 2020; Majumdar et al., 2013; Ragelienė et al., 2022; Ruggiero et al., 2020; Sousa et al., 2020), or winning badges (Bell et al., 2018; Benavides et al., 2021; Cullen et al., 2016; DeSmet et al., 2017; Nollen et al., 2014; Thompson et al., 2015). Many DBDIs also incorporated ≥1 BCT from the ‘*Goals and planning*’ cluster (*n* = 24/41, 59%), including ‘*Goal setting (behavior)’* (*n* = 15/41, 37%), where participants set specific nutrition behavior-related goals in a game-environment (Baranowski et al., 2011; Baranowski et al., 2019; Majumdar et al., 2013; Wang et al., 2015), apps (Nollen et al., 2014), websites (Chamberland et al., 2017; Ezendam et al., 2012), or virtual simulations (Byrne et al., 2012).

### Outcomes assessed

#### Outcome categories

DBDIs were evaluated on primary, secondary, and other outcomes, categorized into three categories: *health outcomes*, *behavior change outcomes*, and *process evaluation outcomes*. *Health outcomes* were assessed in 10 studies (20%) and included mostly anthropometric measures (Baranowski et al., 2011; Baranowski et al., 2019; Bell et al., 2018; Benavides et al., 2021; Chamberland et al., 2017; Chen et al., 2011; Ezendam et al., 2012; Mauriello et al., 2010; Nollen et al., 2014; Whittemore et al., 2013). Among these, all measured BMI pre- and post-intervention. One of these studies (2%) also assessed biochemical measures; Baranowski et al. (2019) evaluated if their game could alter fasting insulin (Baranowski et al., 2019). Most studies assessed *behavior change outcomes* (*n* = 44, 86%), predominantly focusing on food intake (*n* = 33/51, 65%) (Ahn et al., 2016; Baranowski et al., 2011; Baranowski et al., 2019; Bell et al., 2018; Byrne et al., 2012; Chamberland et al., 2017; Chang et al., 2022; Chen et al., 2011; Cullen et al., 2013; Cullen et al., 2016; De Cock et al., 2018; Espinosa-Curiel et al., 2020; Espinosa-Curiel et al., 2022; Ezendam et al., 2012; Farrow et al., 2019; Folkvord et al., 2021; Fraticelli et al., 2016; Hermans et al., 2018; Kato-Lin et al., 2020; Mack et al., 2020; Majumdar et al., 2013; Mauriello et al., 2010; Merino-Godoy et al., 2022; Nollen et al., 2014; Pedersen et al., 2016; Ragelienė et al., 2022; Rosi et al., 2015; Sharma et al., 2015; Shukri et al., 2017; Shukri et al., 2019; Silva et al., 2015; Thompson et al., 2015; Whittemore et al., 2013), knowledge (*n* = 16/51, 31%) (Chang et al., 2022; Chen et al., 2011; De Cock et al., 2018; dos Santos Chagas et al., 2020; Espinosa-Curiel et al., 2020; Espinosa-Curiel et al., 2022; Fraticelli et al., 2016; Froome et al., 2020; Hermans et al., 2018; Mack et al., 2020; Marchetti et al., 2015; Ragelienė et al., 2022; Ruggiero et al., 2020; Schneider et al., 2012; Sharma et al., 2015; Shukri et al., 2019), physical activity (*n* = 13/51, 26%) (Baranowski et al., 2011; Baranowski et al., 2019; Benavides et al., 2021; Cullen et al., 2013; Ezendam et al., 2012; Mack et al., 2020; Majumdar et al., 2013; Mauriello et al., 2010; Pedersen et al., 2016; Sharma et al., 2015; Silva et al., 2015; Whittemore et al., 2013), attitudes (*n* = 9/51, 18%) (Alblas et al., 2020; Benavides et al., 2021; Byrne et al., 2012; de Sousa et al., 2020; Folkvord et al., 2021; Schneider et al., 2012; Sharma et al., 2015; Shukri et al., 2017; Shukri et al., 2019), self-efficacy (*n* = 9/51, 18%) (Bell et al., 2018; Benavides et al., 2021; Byrne et al., 2012; Chen et al., 2011; dos Santos Chagas et al., 2020; Ragelienė et al., 2022; Schneider et al., 2012; Sharma et al., 2015; Whittemore et al., 2013), sedentary behavior (*n* = 7/51, 14%) (Ezendam et al., 2012; Mack et al., 2020; Majumdar et al., 2013; Mauriello et al., 2010; Nollen et al., 2014; Silva et al., 2015; Whittemore et al., 2013), and intention (*n* = 6/51, 12%) (Benavides et al., 2021; de Sousa et al., 2020; Espinosa-Curiel et al., 2022; Ruggiero et al., 2020; Shukri et al., 2017; Shukri et al., 2019). For example, Folkvord et al. (2021) examined changes in children’s eating behavior and attitudes towards healthy and unhealthy foods, as well as their attitude towards the game (Folkvord et al., 2021). *Process evaluation outcomes* were assessed in 30 of experimental studies (59%), and included mostly acceptability (*n* = 18/51, 35%) (Baranowski et al., 2011; Brown et al., 2020; Caon et al., 2022; Champion et al., 2020; Cullen et al., 2013; De Cock et al., 2018; de Sousa et al., 2020; Espinosa-Curiel et al., 2022; Folkvord et al., 2021; Fraticelli et al., 2016; Maes et al., 2011; Marchetti et al., 2015; Ragelienė et al., 2022; Ruggiero et al., 2020; Schneider et al., 2012; Thompson et al., 2015; Thornton et al., 2021; Whittemore et al., 2013), usability (*n* = 8/51, 16%) (Martin et al., 2020; Nollen et al., 2014; Ragelienė et al., 2022; Sharma et al., 2015; Silva et al., 2015; Thompson et al., 2015; Thornton et al., 2021; Whittemore et al., 2013), program experience (*n* = 6/51, 12%) (Chamberland et al., 2017; Cullen et al., 2013; Pedersen et al., 2016; Ragelienė et al., 2022; Sharma et al., 2015; Silva et al., 2015), and feasibility (*n* = 4/51, 8%) (Caon et al., 2022; Espinosa-Curiel et al., 2022; Maes et al., 2011; Martin et al., 2020). For instance, Whittemore et al. (2013) assessed program experience, usage and satisfaction, in addition to its effects on energy balance behaviors (Whittemore et al., 2013). Outcomes from the three categories were mostly shortly assessed after interaction with DBDI. For most experimental studies (*n* = 40, 78%), effects were evaluated within 1-month post-intervention (*n* = 23/40, 58%) (Ahn et al., 2016; Alblas et al., 2020; Bell et al., 2018; Benavides et al., 2021; Byrne et al., 2012; Chang et al., 2022; Cullen et al., 2013; De Cock et al., 2018; dos Santos Chagas et al., 2020; Espinosa-Curiel et al., 2022; Farrow et al., 2019; Folkvord et al., 2021; Fraticelli et al., 2016; Froome et al., 2020; Hermans et al., 2018; Kato-Lin et al., 2020; Mack et al., 2020; Majumdar et al., 2013; Merino-Godoy et al., 2022; Pedersen et al., 2016; Sharma et al., 2015; Silva et al., 2015; Vepsäläinen et al., 2022). Seventeen studies (43%) assessed effects on medium- (≤3 months) (Baranowski et al., 2011; Baranowski et al., 2019; Chamberland et al., 2017; Cullen et al., 2016; DeSmet et al., 2017; Maes et al., 2011; Nollen et al., 2014; Ragelienė et al., 2022; Shukri et al., 2017; Thompson et al., 2015) or long-term (≥6 months) (Caon et al., 2022; Chen et al., 2011; Ezendam et al., 2012; Mauriello et al., 2010; Shukri et al., 2019; Sousa et al., 2020; Whittemore et al., 2013).

#### Favorability of outcomes

Of the 40 included experimental studies, 11 reported no favorable effects of DBDIs (Alblas et al., 2020; Baranowski et al., 2011; Baranowski et al., 2019; De Cock et al., 2018; Ezendam et al., 2012; Folkvord et al., 2021; Hermans et al., 2018; Merino-Godoy et al., 2022; Nollen et al., 2014; Pedersen et al., 2016; Shukri et al., 2017), while 29 papers found some favorable effects on dietary behavior or health outcomes (Table 1). Among these, 23 reported inconsistent findings, i.e. changes in favor of DBDI were not observed for all outcomes (Ahn et al., 2016; Bell et al., 2018; Byrne et al., 2012; Caon et al., 2022; Chang et al., 2022; Cullen et al., 2013; dos Santos Chagas et al., 2020; Froome et al., 2020; Mack et al., 2020; Majumdar et al., 2013; Ragelienė et al., 2022; Sharma et al., 2015; Silva et al., 2015; Sousa et al., 2020; Thompson et al., 2015; Whittemore et al., 2013), or did not maintain at follow-up assessments (Chamberland et al., 2017; Chen et al., 2011; Cullen et al., 2016; DeSmet et al., 2017; Maes et al., 2011; Mauriello et al., 2010; Shukri et al., 2019). Conversely, the remaining 6 papers documented positive effects during both the intervention phase and, where applicable, subsequent follow-up assessment (Benavides et al., 2021; Espinosa-Curiel et al., 2022; Farrow et al., 2019; Fraticelli et al., 2016; Kato-Lin et al., 2020; Vepsäläinen et al., 2022). The game by Espinosa-Curiel et al. (2022), for example, showed to increase knowledge scores and intention to perform healthy behaviors (Espinosa-Curiel et al., 2022) and Vepsäläinen et al. (2022) showed positive results for increasing fruit and vegetable acceptance scores among preschoolers (Vepsäläinen et al., 2022).

Among the included experimental studies (*n* = 40, 78%), studies categorized as ‘*not favorable’* included on average 6.5 (SD = 2.5) BCTs. Studies showing ‘*some favorable outcomes’* integrated a mean of 6.3 (SD = 3.9) BCTs and studies reporting ‘*favorable’* outcomes on average 5.7 (SD = 3.9) BCTs.

## Discussion

This systematic scoping review provides a comprehensive overview of 51 studies utilizing 41 unique DBDIs to promote healthier dietary habits among children and adolescents. It covers technological delivery modes, design and development approaches, behavioral theory, and outcomes assessed, providing a holistic understanding of the DBDI field. It emphasizes diverse study designs and the integration of BCTs to promote and sustain positive behavior change.

### Technological delivery modes

We identified a range of different technologies used in DBDIs, encompassing app-based, web-based, computer-based, or a combination of multiple technologies. Apps were the most common, reflecting the widespread use of smartphones for internet access (Anderson & Teens, 2018). This aligns with many reviews focused on app-based interventions in digital health (Arthurs et al., 2022; Brown et al., 2022; Burrows et al., 2015; Dute et al., 2016; Milne-Ives et al., 2020; Schoeppe et al., 2017; 2016; Yau et al., 2022). The prevalence of app-based approaches in our review (2010–2022) likely reflects the increasing popularity and accessibility of apps in recent years. In contrast, an older broad review (2008–2015) found mostly computer-based interventions and only one app-based DBDI (Lappan et al., 2015), underscoring the rapid deployment of app technology. Regular reviews are needed to track the use of novel technologies, including virtual reality and AI-driven personalized interventions.

### Design and development

Children and adolescents from lower socio-economic backgrounds often face barriers to adopting healthier dietary behaviors due to limited access to essential resources (Livingstone et al., 2023), increasing their risk of unhealthy weight gain or obesity (Anselma et al., 2020; Brug et al., 2012). DBDIs offer a promising avenue by being widely accessible and customizable to cultural and linguistic needs (CBS, 2021; Livingstone et al., 2023). However, most identified DBDIs have been designed and evaluated among non-minority children in high income countries (Anselma et al., 2020; Everson-Hock et al., 2013; Livingstone et al., 2023; Mackert et al., 2016; Oh et al., 2022; Yau et al., 2022), as also reflected in our sample. This underscores the pressing need for research on intervention strategies tailored to diverse contexts (Taj et al., 2019). Future DBDI innovation should prioritize inclusion of diverse populations of children in the design and implementation phases (Livingstone et al., 2023). This may involve soliciting direct input from underrepresented families or studying available DBDIs among children from diverse socio-economic contexts (Livingstone et al., 2023; Nour et al., 2016).

Including co-design approaches into intervention design can enhance engagement and usefulness for end-users by revealing individual needs and wishes (Thabrew et al., 2018). Co-design may particularly hold promise for children from diverse backgrounds such as lower socio-economic status, involving children and key stakeholders in the development and implementation process, ensuring cultural sensitivity, linguistic appropriateness, and resonance with their experiences. This collaborative approach can lead to more engaging, sustainable, and effective interventions (Tay et al., 2021), addressing health inequalities between children from higher and lower socio-economic status (Boyce & Brown, 2017; Livingstone et al., 2023). However, only two DBDIs in our review applied a true co-design process, indicating potential for broader adoption to enhance usability, engagement, and long-term success (Slattery et al., 2020) by cultivating a sense of ownership and connection (Thabrew et al., 2018). However, involving children as co-designers poses challenges related to their cognitive and emotional development and sustained engagement (Thabrew et al., 2018). Future studies should explore strategies to stimulate children’s creative potential for co-creating meaningful dietary interventions.

### Behavioral theory: application in practice

In our sample, all DBDIs applied at least one BCT, but less than half reported using BCTTv1 in-text, posing a challenge for coding (Kok et al., 2016; Peters et al., 2015). On average, DBDIs included 6.2 BCTs, consistent with previous reviews for both children and adults (Direito et al., 2014; Ronteltap et al., 2022; Schoeppe et al., 2017). While techniques varied, most included at least one BCT from the clusters ‘*Feedback and monitoring’, ‘Reward and threat’*, and ‘*Goals and planning*’, aligning with previous reviews (Altenburg et al., 2016; Anselma et al., 2020; Ashton et al., 2019; Brown et al., 2022; Jones et al., 2021; Martin et al., 2013; Schoeppe et al., 2017; Whatnall et al., 2021). Future research should investigate the specific mechanisms underlying these prominent BCTs for promoting dietary behavior change among children (Carey et al., 2019). However, identifying the most effective BCTs, particularly in combinations, and their mechanisms of action (Carey et al., 2019), is complex due to limited robust RCTs and insufficient in-depth reporting of BCTs in DBDIs. As pointed out by Milne-Ives and colleagues (2020), establishing a method for evaluating the confidence level of BCT effectiveness is imperative for systematically developing DBDIs that achieve sustainable long-term improvements in dietary behaviors (Michie et al., 2018; Milne-Ives et al., 2020).

### Outcomes assessed

**Figure 4.**
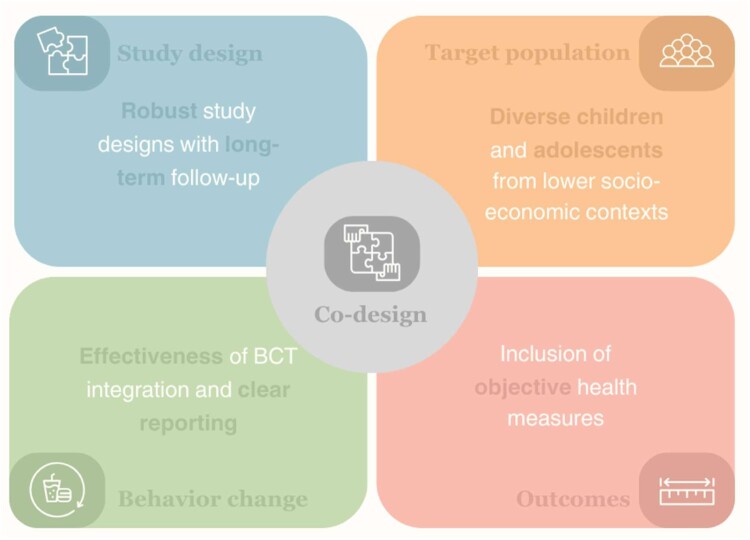
Summary of recommendations for future research. Future research in the field of digital behavioral dietary interventions for dietary behavior change among children should focus on:

DBDIs in this review were evaluated across a diverse range of outcomes, primarily focusing on the outcome categories of behavior change outcomes and process evaluation outcomes. Short-term evaluations generally showed significant changes, while longer-term evaluations (≥6 months) showed mixed effects, possibly due to diminishing DBDI use post-intervention (Borenstein et al., 2010). This diversity in outcomes measured is also observed in other reviews (Kracht et al., 2021). Assessments of health measures were scarce, especially with short follow-up durations (≤3 month) in over three-quarters of the experimental studies, highlighting the need for longer-term assessments to comprehensively evaluate impact (Iribarren et al., 2021; Oikonomidi et al., 2019; Van Rhoon et al., 2020). Future studies should extend follow-up assessments, include objective health measures, and monitor DBDI engagement over time (McNellis et al., 2015; Wu et al., 2019), particularly in children who may need more captivating approaches (Markopoulos et al., 2021). This highlights the critical role of longer-term assessments in revealing effects on behavior and identifying intervention elements contributing to their (in)effectiveness (Oikonomidi et al., 2019). A summary of recommendations for future studies in the field of DBDI development and evaluation among children and adolescents is depicted in Figure 4.

### Strengths and limitations

This review is the first to comprehensively explore DBDIs for dietary behavior change among healthy children and adolescents, incorporating diverse modes of delivery, outcome measures, study designs, and BCT extraction. An important strength is its systematic approach, following PRISMA guidelines, with independent screening and BCT coding by two researchers. Limitations include potential omissions due to the broad search terminology, exclusion of conference papers and/or proceedings reporting recent developments, and heterogeneity in study designs hindering quantitative analysis, and comparison of intervention effectiveness. Moreover, our categorization approach, while useful for organizing the data, may oversimplify nuanced findings and does not account for variations in the methodological quality of included studies, potentially influencing the reliability of our (proxy of) effectivity assessments. Future studies should focus on homogeneous samples for meta-analyses to better capture DBDI effectiveness. Furthermore, we only coded DBDIs without reported BCTs from BCTTv1, potentially overlooking integrated BCTs. Lastly, some theory-based behavioral change intervention elements could not be linked to the BCTTv1 and were not coded, leading to a potentially incomplete overview of DBDIs with a theoretical basis (Anselma et al., 2020).

## Conclusion

The aim of this scoping review was to provide a comprehensive overview of DBDIs aimed at promoting dietary behavior change among children and adolescents, focusing on technological delivery modes, design and development approaches, behavioral theory, and outcomes assessed. Although the field has progressed in recent years, there is a need for more robust study designs in long(er)-term interventions, particularly involving more diverse pediatric populations like those from lower socio-economic backgrounds. Additionally, there is a need for further exploration of the effectiveness of different combinations of BCTs. Collaborating with children and key stakeholders’ allows for the customization of DBDIs to unique contexts, needs, and wishes, potentially decreasing health inequalities. Therefore, co-designing DBDIs represents a promising avenue for developing long-term, tailored, and engaging interventions that may be more effective in promoting dietary behavior change.

## Supplementary Material

Supp 2 Search strategy.docx

Supp 1 PRISMA Checklist.docx

## Data Availability

The dataset generated and analyzed with this scoping review is available upon request.
