# Supplementary material for: Digital behavioral dietary interventions to promote a healthy diet among children and adolescents: a scoping review of technologies, design, behavioral theory, and assessed outcomes
Source: Health Psychol Behav Med. 2024 Nov 29;12(1):2430965. doi: 10.1080/21642850.2024.2430965 (PMC11610228; doi:10.1080/21642850.2024.2430965)
Supplement: Supp 2 Search strategy.docx [file RHPB_A_2430965_SM5908.docx]

# Search strategy

### *Please note that the initial search strategy presented in this document was designed to search for digital behavioral interventions in the fields of nutrition, physical activity, and sedentary behavior. After title and abstract screening, we narrowed the focus to nutrition-related studies to align with our core expertise. Consequently, articles related to physical activity and sedentary behavior were omitted. The search strategy detailed in this document reflects our original search, which included terms for physical activity and sedentary behavior.*

### Objective

The aim of this systematic literature review is to provide a comprehensive overview of currently available digital behavioral dietary interventions (DBDIs) for, to identify the integrated BCTs, and to summarize DBDIs potential effectiveness in changing dietary behavior, physical activity, sedentary behavior, obesity indices and clinical health parameters.

#### Definitions

The following definitions are used in this review:

**Digital behavioral dietary intervention**
A DBDI is defined as software available on any digital device (e.g., tablet, computer, or smartphone) designed to promote healthy behaviors through automated technology-driven interventions.

**Automated**

Automated is defined as functioning independently of human interference.

**Healthy children**

Children are considered healthy if they do not suffer from diseases or conditions that may affect dietary intake (e.g., anorexia nervosa, type 1 diabetes, or cancer). Children with overweight are included, but those with extreme overweight or obesity (>85th percentile) are excluded due to their different nutritional needs, including a lower-calorie diet.

### Research questions

The following research questions are aimed to address:

1. What digital tools are currently available for improving lifestyle among children and adolescents?
2. What is their (potential) effect on improving nutrition behavior, physical activity, sedentary behavior, weight status and health among children and adolescents?
3. Do these digital tools incorporate Behavioral Change Techniques (BCTs), and if so, which are applied?

### Search inclusion and exclusion criteria

|  | Included | Excluded |
| --- | --- | --- |
| Publication type | - Articles published in peer-reviewed journals | - Duplicates - (Systematic) reviews - Meta-analyses - Non-peer-reviewed publications - Grey literature - Books or book chapters - Conference proceedings |
| Language | - English | - Articles not in English |
| Dates | - Published in or after 2010 | - Published before 2010 |
| Target population | - Targeted at healthy children or adolescents (4-18 years old) - Targeted at children or adolescents (4-18 years old) for which the same health guidelines apply as for average children (e.g., overweight or obese children) | - Targeted at children suffering from a disease not influenced by lifestyle (e.g. anorexia nervosa or type 1 diabetes) - Targeted at relatives of children - Targeted at relatives and children |
| Study focus | Digital behavioral dietary intervention   - Studies focusing on an application, tablet-based program, website/web-based program, computer-based-program, chatbot or (video)game - Studies using a text-messaging program - Automated (i.e., no human interference) | Digital behavioral dietary intervention   - Studies focusing on e-books, social media, YouTube, marketing or advertising - Studies focusing on telehealth - Studies focusing on exergaming (i.e., active gaming), executive function, dynamic balance or muscle building skills - Studies that evaluate a digital tool that is not initially developed for the purpose of lifestyle coaching (e.g., Pokémon Go), but developed for commercial purposes |
|  | Lifestyle and health   - Studies focused on changing nutrition behavior (e.g., food intake, food habits, acceptance, knowledge or preference), physical activity, sedentary behavior, obesity indices (e.g., weight, BMI, waist circumference or body fat) and clinical health outcomes (e.g., blood glucose, blood pressure, cholesterol, blood hemoglobine, urine volume/sodium) as a result from using the digital tool - Studies that use multiple interventions/combine interventions | Lifestyle and health   - Studies that do not focus on changing nutrition behavior, physical activity, sedentary behavior, weight status and health outcomes - Studies focused on tabacco, alcohol or other (medical) drugs - Studies focused on mental health (e.g. grief or attention-span) |
|  | Evaluation   - Studies that evaluated change in nutrition behavior, physical activity, sedentary behavior and obesity indices as a result from using the digital tool - Studies that evaluate the usability, acceptability, or feasibility of the tool - Studies that qualitatively evaluate the digital tool | Effect evaluation   - Studies reporting on cost-effectiveness - Studies that only describe the design process, but do not evaluate the tool with end-users |

### Search strategy and steps

1. An initial search strategy (this document) will be developed, defining the parameters of the search that are needed to study the objectives.
2. The search strategy will be adjusted based on preliminary literature research, to ultimately reach a manageable number of hits which are as relevant as possible.
3. The search strategy will be sent to a WUR librarian for refinement.
4. The following databases will be used for the search:
   1. Scopus
   2. PubMed
   3. PsycINFO
5. Screening will be performed in the following steps:
   1. Title screening
   2. Abstract screening
   3. Full article screening
   4. At each stage the team will be updated to avoid possible discrepancies

#### Supplementary/hand searches

The reference list of comprehensive reviews will be searched for additional relevant literature.

## Scopus

### Search blocks and keywords

| Number | Concept | Scopus Query | Results |
| --- | --- | --- | --- |
| 1 | Children | TITLE (child OR children* OR schoolchild* OR childhood OR kid OR kids OR pediatric OR pediatrics OR paediatric OR paedriatics OR youngster* OR youth OR adolescen* OR teenag* OR teen OR teens OR boy OR boys OR son OR sons OR boyhood OR daughter OR girl OR girls OR girlhood OR juvenile OR prepubescen* OR pre-pubescen* OR preschool* OR “pre-school*” OR puber* OR underage* OR “young people” OR “young adults”) | 1,844,149 |
| 2 | Smartphone | TITLE-ABS-KEY (smartphone* OR “smart phone*” OR iphone* OR i-phone* OR android OR “cell phone*” OR cellphone* OR “cellular phone*” OR “computer handheld*” OR “mobile device*” OR mobile-phone* OR “mobile phone*” OR mobile-based OR “mobile based” OR “handheld system*” OR “mobile health tool*” OR mhealth OR m-health OR “mobile health intervention*”) | 256,858 |
| 3 | Computer | TITLE-ABS-KEY (computers OR computer-based OR “computer software” OR computer-assisted OR “technological device*”) | 4,969,288 |
| 4 | Application | TITLE-ABS-KEY (app OR apps OR applet* OR application* OR app-based) | 7,204.090 |
| 5 | Tablet | TITLE-ABS-KEY (tablet* OR ipad* OR smartwatch OR “activity tracker”) | 148,438 |
| 6 | Internet | TITLE-ABS-KEY (internet OR internet-based OR web-based OR “World Wide Web” OR internet-delivered) | 800,489 |
| 7 | Chatbot | TITLE-ABS-KEY ((conversational PRE/3 (agent* OR assistant* OR bot*)) OR (virtual PRE/3 (agent* OR assistant* OR bot*)) OR “chat bot*” OR chatbot* OR infobot*) | 14,997 |
| 8 | Game | TITLE-ABS-KEY (videogame* OR game* OR gaming OR gamification OR “interactive multimedia” OR game-based OR exergame*) | 347,101 |
| 9 | Nutrition behavior | TITLE-ABS-KEY (((intake* OR consumption OR eating OR drinking OR snacking) AND (food OR nutrition OR nutritional OR energy OR calorie* OR vegetable* OR fruit* OR fat* OR "sugar sweetened beverage*" OR "sugary drink*" OR salt OR snack* OR sweet OR sweets OR “energy drink” OR salad OR biscuit* OR schoolmeal* OR meal* OR dinner OR lunch OR breakfast OR energy)) OR ("healthy lifestyle" OR "healthy diet" OR "healthy eating" OR "dietary behavior*" OR "dietary behaviour*" OR "healthy food choice*" OR "food habit*" OR "usual food intake*" OR "food choice*" OR "eating habit*" OR "nutritional habit*" OR "eating behaviour*" OR "eating behavior*" OR "obesity prevention behavior*" OR "obesity prevention behaviour*" OR "health coaching" OR "lifestyle coaching" OR “snacking habit*” OR “snacking behavior*” OR “snacking behaviour*” OR “unhealthy eating” OR “unhealthy diet” OR “unhealthy lifestyle”)) | 1,004,043 |
| 10 | Physical activity | TITLE-ABS-KEY (“physical activit*” OR “physical performance” OR walk* OR “physical fitness” OR sport* OR exercis* OR running OR jogging OR “movement behavior*” OR “movement behaviour*” OR “active lifestyle” OR cycling OR skating OR skeelering OR playground OR football OR hockey OR “leisure activit*”) | 1,686,618 |
| 11 | Sedentary behavior | TITLE-ABS-KEY (sedentary OR sitting OR inactiv* OR “screen time” OR inactivit* OR chilling) | 248,765 |
| 12 | Obesity indices | TITLE-ABS-KEY (obesity OR “weight reduction” OR “weight status” OR “body weight change*” OR “changing weight” OR “weight gain” OR overweight OR “body mass index” OR BMI OR “body fat” OR “waist circumference” OR adipos*) | 1,094,945 |
| 13 | Clinical health outcomes | TITLE-ABS-KEY (“blood pressure” OR “blood glucose” OR “blood hemoglobin” OR “hemoglobin content” OR “blood glucose” OR “hemoglobin concentration” OR “hemoglobin count” OR cholesterol OR “urine volume” OR “sodium test” OR “urinary sodium”) | 2,221,524 |
| 14 | Effect evaluation | TITLE-ABS-KEY (valid* OR effect* OR evaluat* OR “randomized controlled trial” OR “randomised controlled trial” OR RCT OR “clinical trial” OR usage OR usab* OR feasib* OR intervention OR efficacy OR “behavior change” OR “behaviour change” OR “behavioral change” OR “behavioural change” OR functionality OR assessment OR impact OR engagement OR (Lifestyle W/3 (change OR modification OR adaptation)) | 6,560,477 |
| 15 | Effect of digital tools on nutrition behavior | 1 AND (2 OR 3 OR 4 OR 5 OR 6 OR 7 OR 8) AND 9 AND 14 AND PUBYEAR > 2009 | 2,787 |
| 16 | Effect of digital tools on physical activity | 1 AND (2 OR 3 OR 4 OR 5 OR 6 OR 7 OR 8) AND 10 AND 14 AND PUBYEAR > 2009 | 7,373 |
| 17 | Effect of digital tools on sedentary behavior | 1 AND (2 OR 3 OR 4 OR 5 OR 6 OR 7 OR 8) AND 11 AND 14 AND PUBYEAR > 2009 | 2,103 |
| 18 | Effect of digital tools on obesity indices | 1 AND (2 OR 3 OR 4 OR 5 OR 6 OR 7 OR 8) AND 12 AND 14 AND PUBYEAR > 2009 | 4,420 |
| 19 | Effect of digital tools on clinical health outcomes | 1 AND (2 OR 3 OR 4 OR 5 OR 6 OR 7 OR 8) AND 12 AND 14 AND PUBYEAR > 2009 | 1,689 |
| 20 | Total | 1 AND (2 OR 3 OR 4 OR 5 OR6 OR 7 OR 8) AND (9 OR 10 OR 11 OR 12 OR 13) AND 14 AND PUBYEAR >2009 | 9,903 |

#### NOT-terms

AND NOT TITLE-ABS-KEY(“eating disorder” OR anorexia OR “anorexia nervosa” OR “disordered eating” OR tobacco OR cannabis OR alcohol OR sleep OR grief OR “attention span” OR “type 1 diabetes” OR “gestational diabetes” OR autism OR vaccin* OR depress* OR hyperactivity OR ADHD OR gamble OR gambling OR garden* OR cancer OR mindfulness OR “oral health”

**Total: 7,684 results**

## PubMed

### Search blocks and keywords

| Number | Concept | PubMed Query | Results |
| --- | --- | --- | --- |
| 1 | Children | "Child, Preschool"[Majr] OR "Child"[Majr] OR "Adolescent"[Majr] OR child*[ti] OR kid[ti] OR kids[ti] OR pediatric[ti] OR pediatrics[ti] OR paediatric[ti] OR paediatrics[ti] OR youngster*[ti] OR youth[ti] OR adolescent*[ti] OR teenag*[ti] OR teen*[ti] OR boy[ti] OR boys[ti] OR son[ti] OR sons[ti] OR boyhood[ti] OR daughter*[ti] OR girl[ti] OR girls[ti] OR girlhood[ti] juvenile[ti] OR preschool*[ti] OR pre-school*[ti] OR puber*[ti] OR schoolchild*[ti] OR underage*[ti] OR "young people"[ti] OR "young adult*"[ti] | 89,007 |
| 2 | Smartphone | "Smartphone"[Majr] OR "Cell Phone"[Majr] OR smartphone*[tiab] OR “smart phone*”[tiab] OR iphone*[tiab] OR i-phone*[tiab] OR android[tiab] OR "cell phone*"[tiab] OR “cellular phone”[tiab] OR "computer handheld*"[tiab] OR "mobile device*"[tiab] OR mobile-phone*[tiab] OR “mobile phone”[tiab] OR mobile-based[tiab] OR “mobile based”[tiab] OR "handheld system*"[tiab] OR "mobile health tool*"[tiab] OR mhealth[tiab] OR m-health[tiab] OR “mobile health intervention*”[tiab] | 45,606 |
| 3 | Computer | "Computers"[Majr] OR computers[tiab] OR computer-based[tiab] OR computer-based[tiab] OR “computer software”[tiab] OR "technological device*"[tiab] | 70,258 |
| 4 | Application | "Mobile Applications"[Majr] OR app[tiab] OR apps[tiab] OR applet*[tiab] OR application*[tiab] OR app-based[tiab] | 1,444,157 |
| 5 | Tablet | "Computers, Handheld"[Majr] OR tablet*[tiab] OR tablet-based*[tiab] OR "tablet comput*"[tiab] OR "tablet device*"[tiab] OR ipad[tiab] OR ipad-based[tiab] OR "ipad based"[tiab] | 67,111 |
| 6 | Internet | "Internet"[Majr] OR internet[tiab] OR internet-based[tiab] OR "internet based"[tiab] OR web-based[tiab] OR "web based"[tiab] OR "world wide web"[tiab] OR internet-delivered[tiab] OR "internet delivered"[tiab] | 126,456 |
| 7 | Chatbot | “conversational agent*”[tiab] OR “conversational assistant*”[tiab] OR “conversational bot*” OR “virtual agent*”[tiab] OR chatbot*[tiab] OR “chat bot*”[tiab] OR infobot*[tiab] | 764 |
| 8 | Game | "Gamification"[Majr] OR "Video Games"[Majr] OR "Games, Recreational"[Majr] OR videogame*[tiab] OR game*[tiab] OR gaming[tiab] OR gamification[tiab] OR "interactive multimedia"[tiab] OR game-based[tiab] OR exergame*[tiab] OR "active videogame*"[tiab] OR "active video game*"[tiab] | 72,579 |
| 9 | Nutrition behavior | "Feeding Behavior"[Majr] OR "Eating"[Majr] OR ((intake*[tiab] OR consumption[tiab] OR eating[tiab] OR drinking[tiab] OR snacking[tiab]) AND (food[tiab] OR nutrition[tiab] OR nutritional[tiab] OR energy[tiab] OR vegetable*[tiab] OR fruit*[tiab] OR fat[tiab] OR fats[tiab] OR "sugar sweetened beverage*"[tiab] OR "sugary drink*"[tiab] OR salt[tiab] OR snack*[tiab] OR sweet[tiab] OR sweets[tiab] OR “energy drink*”[tiab] OR schoolmeal*[tiab] OR "school meal*"[tiab] OR meal*[tiab] OR dinner[tiab] OR lunch[tiab] OR breakfast[tiab] OR salad[tiab] OR biscuit*[tiab]) OR ("healthy lifestyle"[tiab] OR "healthy diet"[tiab] OR "healthy eating"[tiab] OR "dietary behavior*"[tiab] OR "dietary behaviour*"[tiab] OR "healthy food choice*"[tiab] OR "food habit*"[tiab] OR "usual food intake*"[tiab] OR "food choice*"[tiab] OR "eating habit*"[tiab] OR "nutritional habit*"[tiab] OR "eating behaviour*"[tiab] OR "eating behavior*"[tiab] OR "obesity prevention behavior*"[tiab] OR "health coaching"[tiab] OR "lifestyle coaching"[tiab] OR “snacking habit*”[tiab] OR “snacking behavior*”[tiab] OR “snacking behaviour*”[tiab] OR “unhealthy eating”[tiab] OR “unhealthy diet”[tiab] OR “unhealthy lifestyle”[tiab]) | 350,181 |
| 10 | Physical activity | "Exercise"[Majr] OR “physical activit*”[tiab] OR “physical performance” [tiab] OR walk*[tiab] OR “physical fitness” [tiab] OR sport*[tiab] OR exercis*[tiab] OR running[tiab] OR jogging[tiab] OR “movement behavior*”[tiab] OR “movement behaviour*”[tiab] OR “active lifestyle” [tiab] OR cycling[tiab] OR skating[tiab] OR skeelering[tiab] OR playground[tiab] OR football[tiab] OR hockey[tiab] OR “leisure activit*”[tiab] | 762,263 |
| 11 | Sedentary behavior | "Sedentary Behavior"[Majr] OR sedentary[tiab] OR sitting[tiab] OR inactiv*[tiab] OR “screen time” [tiab] OR inactivit*[tiab] OR chilling[tiab] | 389,484 |
| 12 | Obesity indices | "Pediatric Obesity"[Majr] OR "Obesity"[Majr] OR obesity[tiab] OR “weight reduction”[tiab] OR “weight status”[tiab] OR “body weight change*”[tiab] OR “changing weight”[tiab] OR “weight gain”[tiab] OR overweight[tiab] OR “body mass index”[tiab] OR BMI[tiab] OR “body fat”[tiab] OR “waist circumference”[tiab] OR adipos*[tiab] | 682,297 |
| 13 | Clinical health outcomes | “blood pressure”[tiab] OR “blood glucose”[tiab] OR “blood hemoglobin”[tiab] OR “hemoglobin content”[tiab] OR “blood glucose”[tiab] OR “hemoglobin concentration”[tiab] OR “hemoglobin count”[tiab] OR cholesterol[tiab] OR “urine volume”[tiab] OR “sodium test”[tiab] OR “urinary sodium”[tiab] | 642,439 |
| 14 | Effect evaluation | valid*[tiab] OR effect*[tiab] OR evaluat*[tiab] OR “randomized controlled trial”[tiab] OR “randomised controlled trial”[tiab] OR RCT[tiab] OR “clinical trial”[tiab] OR usage[tiab] OR usab*[tiab] OR feasib*[tiab] OR intervention[tiab] OR efficacy[tiab] OR “behavior change”[tiab] OR “behaviour change”[tiab] OR “behavioral change”[tiab] OR “behavioural change”[tiab] OR functionality[tiab] OR assessment[tiab] OR impact[tiab] OR engagement[tiab] OR “lifestyle change”[tiab] OR “lifestyle modification”[tiab] OR “lifestyle adaptation”[tiab] | 12,507,560 |
| 15 | Effect of digital tools on nutrition behavior | 1 AND (2 OR 3 OR 4 OR 5 OR 6 OR 7 OR 8) AND 9 AND 14 | 1,405 |
| 16 | Effect of digital tools on physical activity | 1 AND (2 OR 3 OR 4 OR 5 OR 6 OR 7 OR 8) AND 10 AND 14 | 3,151 |
| 17 | Effect of digital tools on sedentary behavior | 1 AND (2 OR 3 OR 4 OR 5 OR 6 OR 7 OR 8) AND 11 AND 14 | 1,095 |
| 18 | Effect of digital tools on obesity indices | 1 AND (2 OR 3 OR 4 OR 5 OR 6 OR 7 OR 8) AND 12 AND 14 | 2,022 |
| 19 | Effect of digital tools on clinical health outcomes | 1 AND (2 OR 3 OR 4 OR 5 OR 6 OR 7 OR 8) AND 12 AND 14 | 504 |
| 20 | Total | 1 AND (2 OR 3 OR 4 OR 5 OR6 OR 7 OR 8) AND (9 OR 10 OR 11 OR 12 OR 13) AND 14 AND (2010:2022[pdat]) | 3,945 |

##

## PsycInfo

### Search blocks and keywords

| Number | Concept | PsycInfo Query | Results |
| --- | --- | --- | --- |
| 1 | Children | TI child OR children* OR schoolchild* OR childhood OR kid OR kids OR pediatric OR pediatrics OR paediatric OR paedriatics OR youngster* OR youth OR adolescen* OR teenag* OR teen OR teens OR boy OR boys OR son OR sons OR boyhood OR daughter OR girl OR girls OR girlhood OR juvenile OR prepubescen* OR pre-pubescen* OR preschool* OR “pre-school*” OR puber* OR underage* OR “young people” OR “young adults” | 583,167 |
| 2 | Smartphone | MM "Smartphones" OR smartphone* OR “smart phone*” OR iphone* OR i-phone* OR android OR “cell phone*” OR cellphone* OR “cellular phone*” OR “computer handheld*” OR “mobile device*” OR mobile-phone* OR “mobile phone*” OR mobile-based OR “mobile based” OR “handheld system*” OR “mobile health tool*” OR mhealth OR m-health OR “mobile health intervention*” | 5,407 |
| 3 | Computer | computers OR computer-based OR “computer software” OR computer-assisted OR “technological device*” | 19,679 |
| 4 | Application | MM "Mobile Applications" OR app OR apps OR applet* OR application* OR app-based | 33,572 |
| 5 | Tablet | MM "Tablet Computers" OR tablet* OR ipad* OR smartwatch OR “activity tracker” | 1,661 |
| 6 | Internet | MM "Internet" OR internet OR internet-based OR web-based OR “World Wide Web” OR internet-delivered | 31,655 |
| 7 | Chatbot | MM "Conversational Agents" OR (conversational N3 (agent* OR assistant* OR bot*)) OR (virtual N3 (agent* OR assistant* OR bot*)) OR “chat bot*” OR chatbot* OR infobot*) | 403 |
| 8 | Game | MM "Games" OR videogame* OR game* OR gaming OR gamification OR “interactive multimedia” OR game-based OR exergame* | 22,216 |
| 9 | Nutrition behavior | MM "Eating Behavior" OR MM "Healthy Eating" MM "Food Intake" OR ((intake* OR consumption OR eating OR drinking OR snacking) AND (food OR nutrition OR nutritional OR energy OR calorie* OR vegetable* OR fruit* OR fat* OR "sugar sweetened beverage*" OR "sugary drink*" OR salt OR snack* OR sweet OR sweets OR “energy drink*” OR salad OR biscuit* OR schoolmeal* OR meal* OR dinner OR lunch OR breakfast) OR ("healthy lifestyle" OR "healthy diet" OR "healthy eating" OR "dietary behavior*" OR "dietary behaviour*" OR "healthy food choice*" OR "food habit*" OR "usual food intake*" OR "food choice*" OR "eating habit*" OR "nutritional habit*" OR "eating behaviour*" OR "eating behavior*" OR "obesity prevention behavior*" OR "obesity prevention behaviour*" OR "health coaching" OR "lifestyle coaching" OR “snacking habit*” OR “snacking behavior*” OR “snacking behaviour*” OR “unhealthy eating” OR “unhealthy diet” OR “unhealthy lifestyle”) | 107,361 |
| 10 | Physical activity | MM "Physical Activity" OR MM "Actigraphy" OR MM "Exercise" OR “physical activit*” OR “physical performance” OR walk* OR “physical fitness” OR sport* OR exercis* OR running OR jogging OR “movement behavior*” OR “movement behaviour*” OR “active lifestyle” OR cycling OR skating OR skeelering OR playground OR football OR hockey OR “leisure activit*” | 236,122 |
| 11 | Sedentary behavior | MM "Sedentary Behavior" OR sedentary OR sitting OR inactiv* OR “screen time” OR inactivit* OR chilling | 30,921 |
| 12 | Obesity indices | MM "Obesity" OR obesity OR “weight reduction” OR “weight status” OR “body weight change*” OR “changing weight” OR “weight gain” OR overweight OR “body mass index” OR BMI OR “body fat” OR “waist circumference” OR adipos* | 81,910 |
| 13 | Clinical health outcomes | “blood pressure” OR “blood glucose” OR “blood hemoglobin” OR “hemoglobin content” OR “blood glucose” OR “hemoglobin concentration” OR “hemoglobin count” OR cholesterol OR “urine volume” OR “sodium test” OR “urinary sodium” | 37,980 |
| 14 | Effect evaluation | valid* OR effect* OR evaluat* OR “randomized controlled trial” OR “randomised controlled trial” OR RCT OR “clinical trial” OR usage OR usab* OR feasib* OR intervention OR efficacy OR “behavior change” OR “behaviour change” OR “behavioral change” OR “behavioural change” OR functionality OR assessment OR impact OR engagement OR (Lifestyle N3 (change OR modification OR adaptation)) | 2,799,660 |
| 15 | Effect of digital tools on nutrition behavior | 1 AND (2 OR 3 OR 4 OR 5 OR 6 OR 7 OR 8) AND 9 AND 14 | 815 |
| 16 | Effect of digital tools on physical activity | 1 AND (2 OR 3 OR 4 OR 5 OR 6 OR 7 OR 8) AND 10 AND 14 | 1,897 |
| 17 | Effect of digital tools on sedentary behavior | 1 AND (2 OR 3 OR 4 OR 5 OR 6 OR 7 OR 8) AND 11 AND 14 | 599 |
| 18 | Effect of digital tools on obesity indices | 1 AND (2 OR 3 OR 4 OR 5 OR 6 OR 7 OR 8) AND 12 AND 14 | 835 |
| 19 | Effect of digital tools on clinical health outcomes | 1 AND (2 OR 3 OR 4 OR 5 OR 6 OR 7 OR 8) AND 12 AND 14 | 73 |
|  | Total | 1 AND (2 OR 3 OR 4 OR 5 OR6 OR 7 OR 8) AND (9 OR 10 OR 11 OR 12 OR 13) AND 14 AND (2010:2022[pdat]) Limiters – Publication Year 2010-2023 | 2,737 |
